# Hierarchical Virtual Screening Based on Rocaglamide Derivatives to Discover New Potential Anti-Skin Cancer Agents

**DOI:** 10.3389/fmolb.2022.836572

**Published:** 2022-06-02

**Authors:** Igor V.F. dos Santos, Rosivaldo S. Borges, Guilherme M. Silva, Lúcio R. de Lima, Ruan S. Bastos, Ryan S. Ramos, Luciane B. Silva, Carlos H. T. P. da Silva, Cleydson B. R. dos Santos

**Affiliations:** ^1^ Modeling and Computational Chemistry Laboratory, Federal University of Amapá, Macapá, Brazil; ^2^ Graduate Program in Biotechnology and Biodiversity-Network BIONORTE, Federal University of Amapá, Macapá, Brazil; ^3^ Graduate Program in Medicinal Chemistry and Molecular Modeling, Federal University of Pará, Belém, Brazil; ^4^ Computational Laboratory of Pharmaceutical Chemistry, School of Pharmaceutical Sciences of Ribeirão Preto - Universidade de São Paulo, Ribeirão Preto, Brazil; ^5^ Departamento de Química, Faculdade de Filosofia, Ciências e Letras de Ribeirão Preto - Universidade de São Paulo, Ribeirão Preto, Brazil

**Keywords:** hierarchical virtual screening, rocaglamide, skin cancer, anticancer activity, pharmacophore

## Abstract

Skin Cancer (SC) is among the most common type of cancers worldwide. The search for SC therapeutics using molecular modeling strategies as well as considering natural plant-derived products seems to be a promising strategy. The phytochemical Rocaglamide A (Roc-A) and its derivatives rise as an interesting set of reference compounds due to their *in vitro* cytotoxic activity with SC cell lines. In view of this, we performed a hierarchical virtual screening study considering Roc-A and its derivatives, with the aim to find new chemical entities with potential activity against SC. For this, we selected 15 molecules (Roc-A and 14 derivatives) and initially used them in docking studies to predict their interactions with Checkpoint kinase 1 (Chk1) as a target for SC. This allowed us to compile and use them as a training set to build robust pharmacophore models, validated by Pearson’s correlation (*p*) values and hierarchical cluster analysis (HCA), subsequentially submitted to prospective virtual screening using the Molport^®^ database. Outputted compounds were then selected considering their similarities to Roc-A, followed by analyses of predicted toxicity and pharmacokinetic properties as well as of consensus molecular docking using three software. 10 promising compounds were selected and analyzed in terms of their properties and structural features and, also, considering their previous reports in literature. In this way, the 10 promising virtual hits found in this work may represent potential anti-SC agents and further investigations concerning their biological tests shall be conducted.

## Introduction

Cancer is the name given to a set of diseases characterized by disordered or abnormal cell growth. These defective cells may subsequently invade neighboring tissues or organs, spreading throughout the body in form of different types of neoplasms (Balkwill et al., 2012; Zugazagoitia et al., 2016; Salem et al., 2017).

Between 5 and 10% of neoplasms are associated with genetic inheritance related to cancer. Nonetheless, a large part accounts to damage of genetic material provoked by physical, chemical or biological factors, which accumulates throughout life (Hawk and Lippman, 2000; World Cancer Research Fund/American Institute for Cancer Research, 2018).

Skin cancer (SC) is among the most common type of cancers worldwide. It is usually caused by the excessive incidence of UVB radiation and affects specially Caucasians. Among different types of SC, the non-melanoma type is most frequently found. This type of cancer accounts for 90% of all SC, and its incidence has increased mainly among younger people. In addition, there are other types of SC such as melanoma, basal cell carcinoma, and squamous cell carcinoma (Mueller and Reichrath, 2008; Gordon, 2009).

When it comes to evaluating possible biological targets associated with anti-SC action, some hypotheses have been considered useful and valid. For instance, Sarkaria et al. (1999) reported the inhibition of Chk1 as a prominent protein target within this context. Chk1 is a phosphotransferase kinase required for checkpoint signaling in DNA-damaged cells. Furthermore, Chk1 has been found to be overexpressed in a variety of human breast, colon, liver, gastric, and nasopharyngeal carcinomas. Notably, its expression often positively correlates with tumor grade and disease recurrence (Zhang and Hunter, 2014).

Another important target for evaluation of anti-SC agents is the BRAF kinase, which is mutated in most type tumors. Furthermore, clinical trials show that BRAF kinase inhibitors in combination with other MEK kinase inhibitors are among the most promising chemotherapy regimens for the treatment of advanced BRAF mutant melanoma (Fujimura et al., 2019).

In SC, there are oncogenic signaling pathways that converge on eukaryotic initiation factor 4F (eIF4F), which also makes it a prominent target (Grafanaki et al., 2019). This is composed of the cap-binding protein eIF4E, an RNA helicase eIF4A, and a scaffold protein eIF4G (Gingras et al., 1999). In addition, increased eIF4A protein, and others from the same family, are related to poor clinical prognosis (Liang et al., 2014; Robichaud et al., 2014).

**FIGURE 1:**
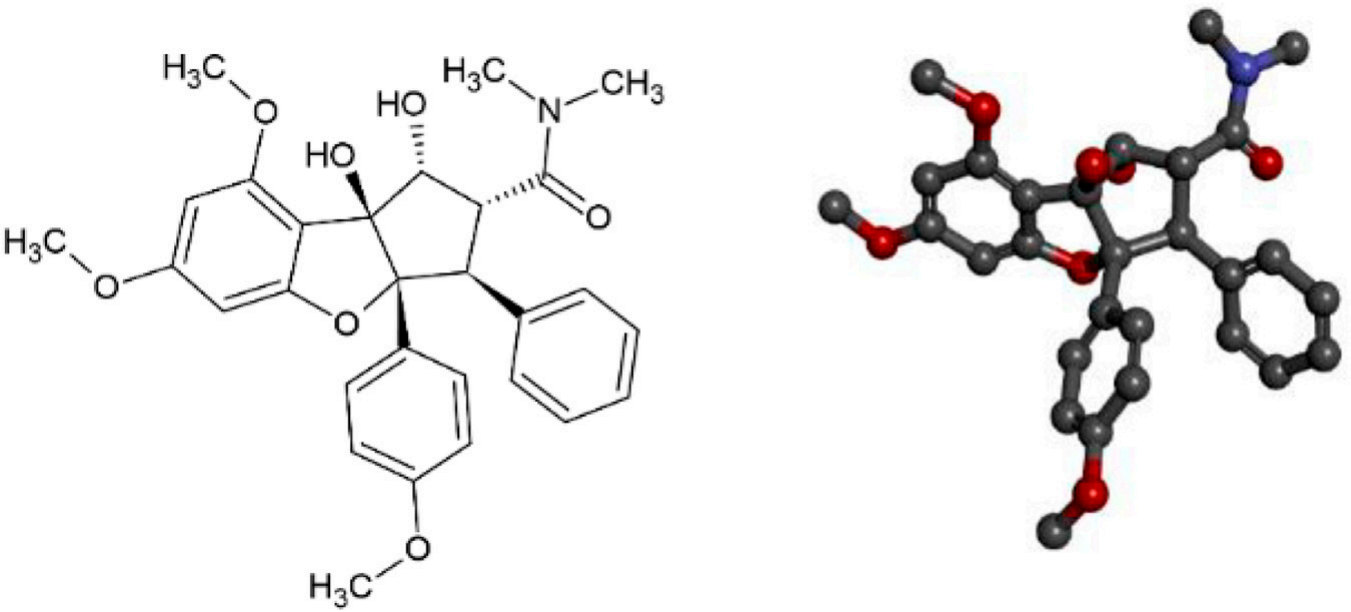
Representation of 2D and 3D structures of Roc-A (1R,2R,3S,3aR,8bS)-1,8b-dihydroxy-6,8-dimethoxy-3a-(4-methoxyphenyl)-N,N-dimethyl-3-phenyl-2,3-dihydro-1H-cyclopenta [b][1]benzofuran-2-carboxamide).

Several anticancer drugs have been discovered by molecular modeling strategies as well as screening of natural plant-derived products (Pezzuto, 1997; Da Rocha et al., 2001). The phytochemical compound Roc-A (Figure 1) belongs to the chemical class of cyclopenta [b]-tetrahydrobenzofurans, collectively called flavaglins or rocaglamides, which are known to kill malignant cancer cells while sparing normal cells (Ebada et al., 2011; Basmadjian et al., 2013; Li-Weber, 2015).

Roc-A and other flavaglins have shown cytotoxic activity based on several *in vitro* experiments using different SC cell lines. For instance, Roc-A presented cytotoxic activity proven in RPMI-7951 cells (IC_50_ = 0.002 μg/ml) and kB cells (IC_50_ = 0.006 μg/ml) (Wu et al., 1997; Basmadjian et al., 2013). Insightfully, some Roc-A derivatives has also shown potential insecticidal activity (Nugroho et al., 1997; Nugroho et al., 1999). Thus, this raises the question whether it would be possible to consider the potential anticancer activity of Roc-A and its derivatives, overcoming any toxicity issues, as a starting point for a molecular modeling study with interest in the treatment of SC.

**FIGURE 2:**
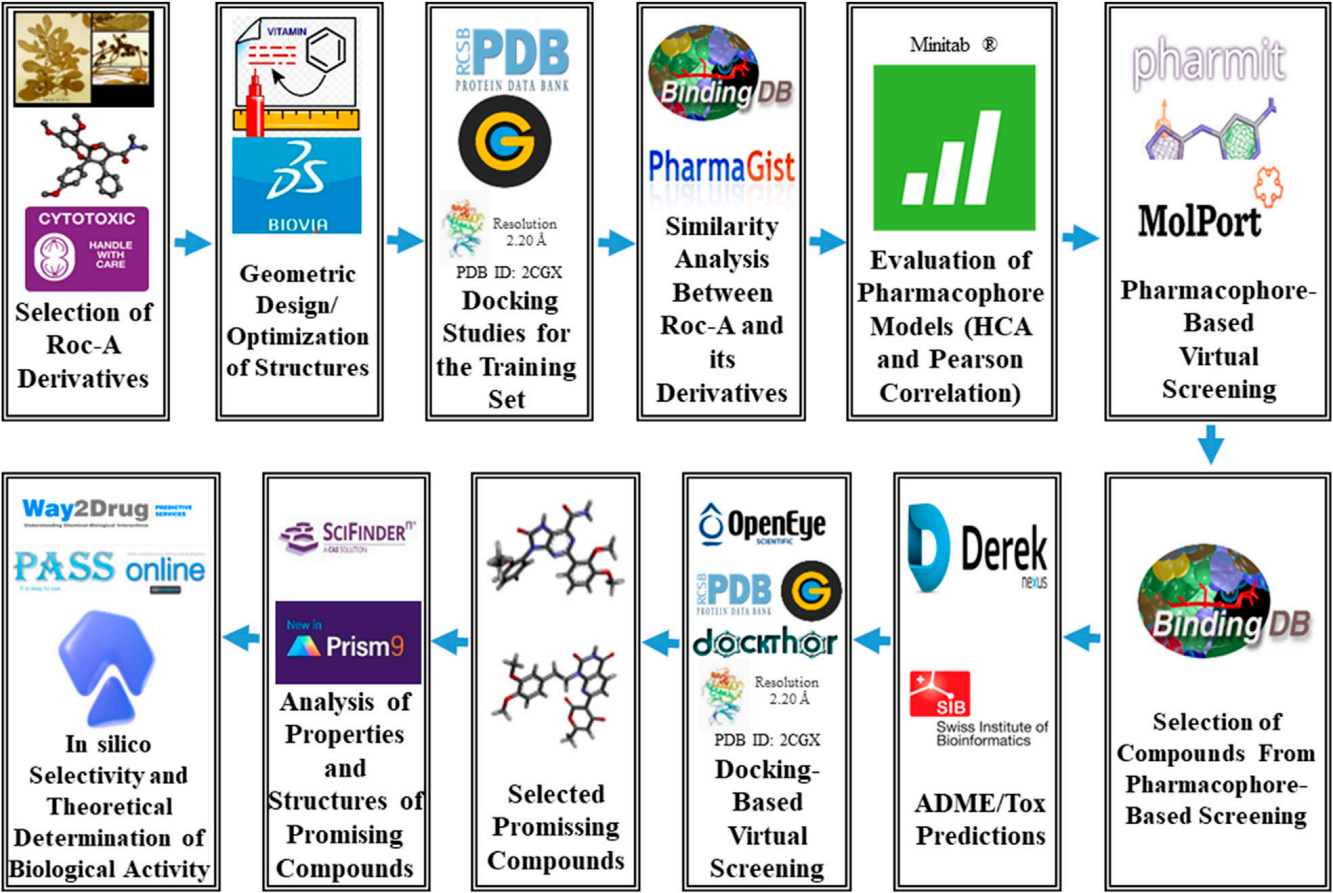
General scheme summarizing the methodological steps proposed via hierarchical virtual screening in this work.

With this in mind, in this study, we sought to perform a study consisting of hierarchical virtual screening to obtain new chemical entities with potential anti-SC activity. For this, we considered available biological information of Roc-A and its derivatives, such as their cytotoxic activity towards SC cell lines and a set of computational methodologies as depicted by the flowchart in Figure 2.

## Materials and Methods

### Selection of Roc-A and Derivatives: Training Set

**FIGURE 3:**
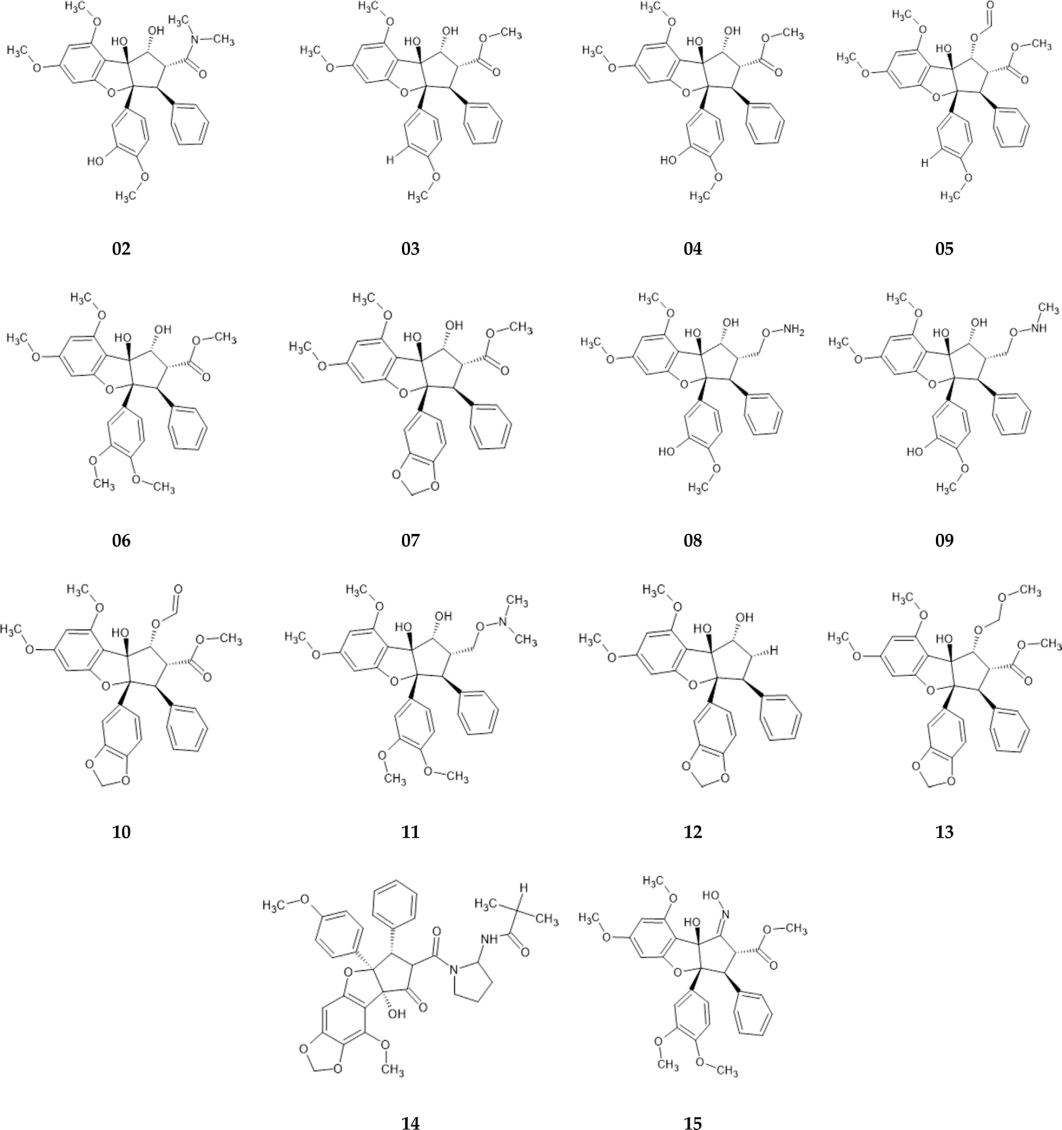
Training set consisting of fourteen Roc-A derivatives, described by Nugroho et al. (1997), Nugroho et al. (1999), used to build pharmacophore models.

Roc-A and its derivatives were selected according to studies of Nugroho et al. (1997), Nugroho et al. (1999), which investigated Roc-A and its derivatives for their potential insecticidal activity. Thus, from this study we selected Roc-A and 14 derivatives, i.e., 15 compounds (Figure 3), which we here denominate as training set.

### Selection of Protein Complex

In order to select the most suitable protein complex of Chk1 to be used in our docking studies we have evaluated different structures retrieved from the Protein Data Bank (PDB, https://www.rcsb.org/). Initial search in such databank retrieved 149 entries, from which 19 were discarded since they presented resolution higher than 2.5 Å. From the remaining, we visually inspected 5 PDB entries which consisted of protein complexes of Chk1 bound to native ligands apparently similar to Roc-A.

In addition, we analyzed the overlap between chemical structures of Roc-A and 5 native ligands within the corresponding binding site of these PDB files. For this, the structural similarities of compounds—in terms if steric and electrostatic features—were assessed by using the software BIOVIA Discovery Studio Visualizer (v 17.2.0.16349) (Biovia et al., 2000).

### Docking Studies for the Training Set

In advance of performing docking simulations, the protein structure of Chk1 (PDB code 2CGX) was prepared by importing it into the Protein Preparation Wizard software (Madhavi Sastry et al., 2013; Schrödinger, 2018), then its pre-processing was done by checking the following functions: assignment of bond orders using CCD (Chemical Component Dictionary) database (Westbrook et al., 2015), addition of hydrogens, generation of disulfide bonds, use of Prime to fill missing loops and side chains, and removal of water molecules; excluding ligands, cofactors and metals. The binding pocket defined in this work for such structure, except when mentioned, was defined by the following centroid (in terms of spatial coordinates): x = 4.9375, y = -5.3174, z = 17.8840. Docking simulations were developed using default settings in GOLD docking software (Verdonk et al., 2003; CCDC, 2015). Therefore, here we employed the CHEMPLP scoring function and a 10 Å sphere radius centered on the mentioned centroid.

In order to validate docking studies redocking simulations were performed using the previously prepared protein from PDB ID 2CGX, considering the above mentioned centroid as well as standard settings of each software. Worth noting that previous to redocking simulations, a simple preparation of the native ligand was carried out, in which such molecule was considered flexible in a 3D format (mol2), with adjustment of the bond orders, addition of hydrogen atoms, and calculation of partial charges.

Prior to docking, the training set was preprocessed using the OMEGA software (Hawkins and Nicholls, 2012; OpenEye, 2020). General parameters were set standard with generation of only 1 minimum energy conformer per molecule; adjustment of the strain energy (above the energy of global minimum conformer) was considered until 9.0 kcal/mol, as well as RMSD (root mean square deviation) of 0.6 Å (root mean square deviation) as a cutoff for conformer identity, as previously reported by us (da Silva and Taft, 2017).

### Similarity Analysis Between Roc-A and Its Derivatives

In advance of building pharmacophore models, structures of 15 compounds were drawn in the ACD/ChemSketch program (freeware) 2020 1.2 (Hunter, 1997) and submitted to geometry optimization in the program BIOVIA Discovery Studio Visualizer (v 17.2.0.16349) (Biovia et al., 2000). The force field used was the MM+ (Molecular Mechanics), according to the methodological strategy proposed by da Silva Costa et al. (2018); afterwards the structures underwent refinement by using the Dreiding-like force field (Hahn, 1995).

After optimization of compounds, their structures were inputted in the BIOVIA Discovery Studio Visualizer (v17.2.0.16349) and gathered into a single file (mol2). Then, this file was submitted to the BindingDB webserver (https://www.bindingdb.org/bind/index.jsp) for calculation of similarities values by means of Tanimoto index (TI) (Liu et al., 2007). TI values (Eq. 1) varies between 0 and 1, representing the overall similarity between two compounds based on their fingerprint bits (molecular fragments), so that the closer to 1, greater the similarity (Gimeno et al., 2019).

$$Tanimoto\;Index=\frac{c}{\left(a+b-c\right)\;}$$ (1)

Where, for two generic compounds A and B: *a*: number of bits in A; *b*: number of bits in B; *c*: number of common bits between A and B.

### Building Pharmacophore Models

The input file with optimized structures of 15 compounds was submitted to the Pharmagist webserver (https://bioinfo3d.cs.tau.ac.il/PharmaGist/) (Schneidman-Duhovny et al., 2008b) to generate pharmacophoric features of Roc-A and its derivatives—considering Roc-A as the pivot molecule. Worth noting that Pharmagist generates pharmacophore models based on the overlap of individual pharmacophoric features of each molecule inputted. Therefore, the method essentially aligns and overlaps the pivot molecule with other molecules from the training set, seeking chemical and spatial characteristics common to the greatest number of molecules. The resulting set with the highest score and the highest number of aligned molecules should be subsequently evaluated to be considered a valid pharmacophore model (Schneidman-Duhovny et al., 2008a; da Silva Costa et al., 2018).

The idea was to select pharmacophore models, constituted by validated pharmacophoric features and alignments, to initiate our hierarchical virtual screening—as in previous studies of Cruz et al. (2018); Ferreira et al. (2019). From this, basically, the aim is to apply such models to identify new compounds, within large and commercial databases, which may show a greater chance of presenting the biological activity of interest as well as appropriate pharmacological properties.

### Evaluation of Pharmacophore Models

From the data obtained using Pharmagist, we constructed a matrix with four main pharmacophoric descriptors/features and their associations with TI values for each compound. This allowed us to calculate Pearson’s correlation values *p*, which measures the degree of relationship between the variables (da Silva Costa et al., 2018; Ferreira et al., 2019). The *p* value has a dimensionless value expressed in the numerical range from −1.0 to +1.0. When the *p* value is equal to 0.0, there is no linear correlation between the analyzed variables. However, general range of values ≤0.2, 0.2 to 0.4, and ≥0.7 indicate weak, moderate, and strong correlations, respectively. A *p* value of +1.0 indicates a perfect positive correlation between the variables; a *p* value of −1.0 indicates a perfect negative correlation between the variables (that is, if one increases the other decreases) (Ferreira et al., 2019).

Hierarchical Cluster Analysis (HCA) was also applied to evaluate the relationship between the pharmacophoric variables. This statistical method can show the similarity (or difference) between descriptors, individually, considering both *p* values and distance methods (Santos et al., 2014; Ferreira et al., 2019). For the construction of the HCA dendrograms and statistical analysis, the Minitab^®^ program was used (Minitab, 2022).

### Pharmacophore-Based Virtual Screening

To employ the pharmacophore models in virtual screening we used the Pharmit^®^ platform (https://pharmit.csb.pitt.edu/), an online tool that uses the state-of-the-art sublinear algorithms to provide an interactive screening of millions of compounds. In addition, the platform offers specific information based on pharmacophore, spatial arrangement of interaction characteristics, molecular formula, and energy minimization (Sunseri and Koes, 2016).

Hence, we initially applied pharmacophore-based virtual screening using its implemented database from the company Molport^®^ (https://www.molport.com/shop/index), which has approximately 8 million molecules. In addition, we applied filters such as maximum and minimum values of physicochemical properties for the studied compounds, by means of the webserver Molinspiration^©^ (https://www.molinspiration.com/), Slovensky Grob, Slovakia. However, the first obtained pharmacophore model, with seven pharmacophoric features, could only retrieve a small number of compounds in initial virtual screening. Therefore, we performed several recombinations amongst the pharmacophoric features in order to achieve/generate more pharmacophore models and obtain a greater number of compounds in virtual screenings, as discussed on results section.

### Selection of Compounds From Pharmacophore-based Screening

Each set of compounds - retrieved from each one of the seven models employed in pharmacophore-based virtual screening - was considered to calculate corresponding similarities (TI values) in relation to Roc-A. TI values were calculated using the webserver BindingDB (https://www.bindingdb.org/bind/index.jsp) (Liu et al., 2007). Then, TI values for compounds were sorted and we selected top 200 from each set, considering a minimum threshold of ≥0.2 for TI values.

### Prediction of Toxicity and Pharmacokinetic Properties

The SwissADME (http://www.swissadme.ch/) is a free webtool that gives access to a set of fast, yet robust, predictive models to estimate: physicochemical properties, pharmacokinetics, drug similarity, and medicinal chemistry compatibility. SwissADME was therefore used to select the most promising compounds considering the following filters: “High” Gastrointestinal Absorption (GIA), “No” Blood Brain Barrier (BBB) Permeation, and “0” Lipinski’s rule violations.

The toxicity profiles of compounds was evaluated using DEREK 10.0.2 software (Nexus, 2011). DEREK (Deductive Estimation of Risk from Existing Knowledge) according to the protocol proposed by (Nexus, 2011; Ferreira et al., 2019). In this way, the following filters were considered: type of toxicity endpoint, description of toxicophoric group, and toxicity alert.

### Docking-Based Virtual Screening

Following the methodological proposal (Figure 2), 60 compounds were retrieved from the pharmacophore-based and toxicity/pharmacokinetic properties screenings. These were submitted to a further screening step to select most promising compounds according to their consensus docking analysis using 3 software. Worth mentioning that we employed same methodological steps as in topic 2.3 to pre-process these 60 compounds as well as same prepared Chk1 protein structure. Thus, docking simulations were performed using default settings in each of the 3 docking software: GOLD (Verdonk et al., 2003; CCDC, 2015), FRED (McGann, 2011; OpenEye, 2020) and DockThor (Guedes et al., 2021). In GOLD, they were carried out in a similar way to the methodological step *Docking Studies for the Training Set*. In FRED, first, spruce4docking (OpenEye, 2020) to process apo structure of Chk1 (2CGX.pdb; prepared as described above), in order to generate “receptor” for a binding pocket and thus indicate a representative residue for such cavity. The compounds were then processed by OMEGA (OpenEye, 2020) to generate 300 conformers for each molecule and docking runs were conducted in standard (default) mode, using its implemented Chemgauss4 scoring function. In DockThor, configurations were employed considering the same binding pocket centroid as in GOLD, and scores were predicted considering the binding affinity (in kcal/mol units) for compounds by the implemented DockTScore program.

### Analysis of Properties and Structures of Promising Compounds

Promising compounds retrieved after docking screening passed through analysis of their lipophilicity and water solubility expressed by means of values of *logP* and *logS*, respectively. This was performed using the prediction software SwissADME (Daina et al., 2017), based on the methodological proposal of Sepay et al. (2020).

SwissADME provides five methods to predict *logP* values: iLOGP, xLOGP3, WLOGP, MLOGP and Silicos-IT. The iLOGP is an internal physical method of SwissADME, based on free solvation energies in *1*-octanol/water and calculated by the Generalized-Born model and solvent accessible surface area (GB/SA) (Daina et al., 2014). xLOGP3 uses known *logP* values from reference compounds as starting point to perform predictions (Cheng et al., 2007). WLOGP is a purely atomistic method based on the fragmentary system of Wildman and Crippen (1999). MLOGP is a standard model of topological method, based on a linear relationship considering 13 molecular descriptors (Moriguchi et al., 1994). Silicos-IT is a hybrid method that has 27 fragments and 7 topological descriptors calculated by the FILTER-IT software—developed by the company SILICOS-IT (http://silicos-it.be.s3-website-eu-west-1.amazonaws.com/index.html).

Also, SwissADME provides 3 topological methods regarding prediction of *logS* values: ESOL, ALI e Silicos-IT. ESOL is a quantitative structure-property relationship (QSPR) model that establishes linear relationships between *logS* and 4 molecular parameters: molar mass, number of rotatable bonds, fraction of aromatic heavy atoms, and xLOGP3 (Delaney, 2004). The model adapted from Ali et al. (2012) relates *logS* to *logP* and TPSA (Topological Polar Surface Area). The company Silicos-IT also offers a method to predict *logS* values considering the FILTER-IT software, and based on a system of 16 fragments modulated by the square root of the molar mass (Daina et al., 2017). Finally, we mention that we used the program GraphPad Prism 9^©^ to build graphics related to these analyses.

In addition, the final promising compounds were submitted to search in the webserver SciFinder^®^ - available for access in the CAS (Chemical Abstract Service) - to obtain information whether their structures were associated with previous studies, or reports regarding their biological activities, following the methodological proposal developed by Ferreira et al. (2019).

Also, a similarity analysis was conducted between the obtained promising compounds and the pivot molecule (Roc-A). For this, we calculated the percentage of steric overlap at 50, 70, and 100% of contribution for each of the final promising compounds in relation to Roc-A using the software Discovery Studio Visualizer (v.17.2.0.16349)—according to the methodological proposal of da Silva Costa et al. (2018) and Cruz et al. (2018).

### *In silico* Evaluation of Selectivity and Theoretical Determination of Biological Activity

After analyzing the properties and structures of the promising compounds, those with the best classified parameters were selected for the molecular docking simulations, following the methodology proposed by Ramos et al. (2019). In this step of the methodology, the values of binding free energy (∆G) related to the interactions of promising compounds from the Pharmacophore-based Virtual Screening will be evaluated, as well as analysis of the binding mode, always comparing results to the control compound Roc-A.

The structures (promising compounds, control compound and molecular targets) used in the study were prepared using the Discovery Studio Visualizer software (v.17.2.0.16349) (Biovia et al., 2000). The receptors Chk1 (PDB ID 2CGX) (Foloppe et al., 2006), elF4A1-ATP (PDB ID 5ZC9) (Iwasaki et al., 2019) e BRAF (PDB ID 6XFP) (Yen et al., 2021), all of the *Homo sapiens* organism, with their respective complexed inhibitors (3D3, RCG and V1Y), were used in the AutoDock 4.2/Vina 1.1.2 software with a graphical interface in the PyRx software version 0.8.30 (https://pyrx.sourceforge.io). The Molecular Docking methodology was validated by calculating the RMSD performed by comparing the conformation of the crystallographic ligand and the best conformation obtained via Molecular Docking.

The coordinates of the Grid center (x, y and z) of the active sites were obtained considering the coordinates of the complexed ligands (see Table 1). In order to evaluate the binding affinity, it was used a binding free energy (∆G) function score derived from the interaction of ligands with amino acid residues of receptors via AutoDock 4.2/Vina 1.1.2 was used. Interaction figures and interaction distance measurements were made using Discovery Studio Visualizer (v.17.2.0.16349) (Biovia et al., 2000). The Heatmap figure was made using the software GraphPadPrism 8.0 (GraphPad Software Inc., San Diego, CA).

**TABLE 1:** Protocol data used for molecular docking validation.

Receptor	Ligand/ID	Coordinates of grid center	Grid box size
Chk1 (*Homo sapiens*) PDB ID: 2CGX	2-[(6-amino-7h-purin-8-yl)thio]acetamide/3D3	X = 4.602	20x
		Y = -5.735	28y
		Z = 17.765	18z
elF4A1-ATP (*Homo sapiens*) (PDB ID: 5ZC9)	(1R,2R,3S,3aR,8bS)-6,8-dimethoxy-3a-(4-methoxyphenyl)-N,N-dimethyl-1,8b-bis(oxidanyl)-3-phenyl-2,3-dihydro-1H-cyclopenta [b][1]benzofuran-2-carboxamide/RCG	X = 42.459	32x
		Y = 5.194	38y
		Z = 44.166	32z
BRAF kinase (*Homo sapiens*) (PDB ID: 6XFP)	4-amino-N-{1-[(3-chloro-2-fluorophenyl)amino]-6-methylisoquinolin-5-yl}thieno [3,2-days]pyrimidine-7-carboxamide/V1Y	X = -3.752	38x
		Y = 15.954	38y
		Z = 13.702	24z

The prediction of in silico biological activity of promising compounds was performed via PASS Online (http://way2drug.com/passonline/). According to the developers’ definition, this is software designed to assess the biological potential of an organic drug-like molecule. This provides prediction of many types of biological activity with average accuracy above 95%. The probability of “to be active” (Pa) and the probability of “to be inactive” (Pi) are estimated by comparison with the molecules of the PASS training set. (Filimonov et al., 2014).

Leave-one-out cross-validation (LOO CV) is performed using the PASS training set for prediction validation the biological spectrum is predicted for each compound using the structure-activity relationship (SAR) as activities from other data for all compounds. Then, the result is compared with known experimental data for the promising compound studied. The procedure is repeated with compounds from the PASS training set; then the mean values of Invariant Prediction Precision (IAP = 1-IEP) are calculated for each biological activity and for all biological activities (Filimonov et al., 2014).

The in silico prediction of cytotoxic effect of promising compounds was performed via CLC-Pred (Cell Line Cytotoxic Predictor) (http://www.way2drug.com/cell-line/). An in silico prediction web-service of cytotoxicity of chemical compounds in untransformed or cancer cell lines. The development of the CLC-Pred was used the PASS algorithm to create and validate the SAR classification models. The CLC-Pred training set contains thousands of compound structures with their experimental data from ChemBL. The average accuracy of the prediction calculated by LOO CV is 93% (Lagunin et al., 2018).

Lagunin et al., 2018 emphasizes that only activities with Pa > Pi are possible for a compound. Furthermore, Pa measures the similarity of the predicted compound with the structures of the compounds, which are the most typical in a subset of active in the web-server training set.

## Results and Discussion

### Selection of Chk1 Protein Complex

The selection of the target Chk1 was based on the work of Lu et al. (2008) and Sarkaria et al. (1999), which mentions it as an important target associated with SC. In fact, Da Silva Costa et al. (2018) also used Chk1 as a target associated with skin cancer in a virtual screening study.

Several protein complexes for Chk1 are found in the PDB, however, the structure of such protein complexed with Roc-A has not been disclosed yet. Therefore, in order to select a reliable Chk1 protein complex to this study, we considered performing chemical-similarity comparisons between Roc-A and native ligands of corresponding protein-ligand complexes available in the PDB.

**FIGURE 4:**
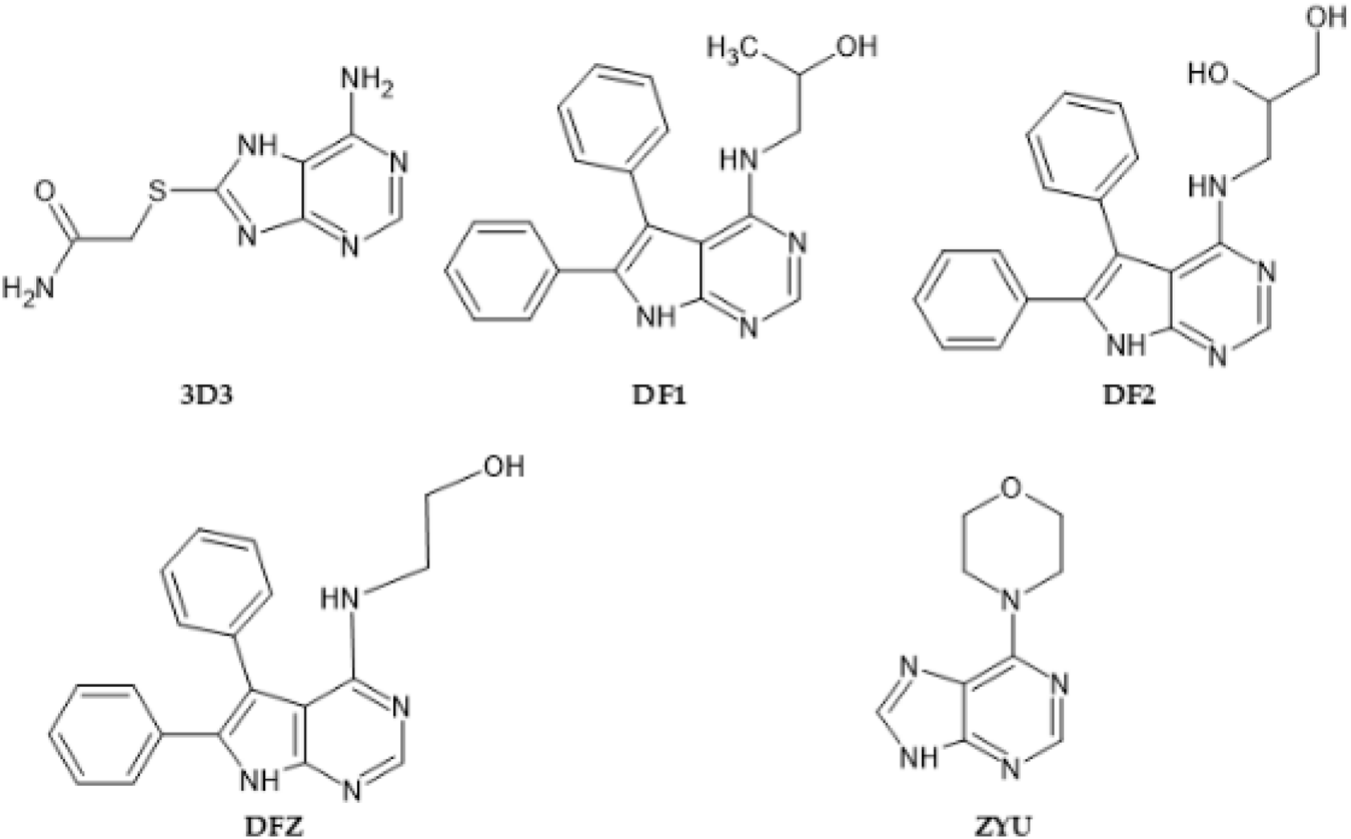
2D structures of native ligands complexed with Chk1 protein structures retrieved from PDB.

In this manner, as described in methods section, we selected 5 native ligands (from 5 different protein-ligand complexes of Chk1) to compare them with the structure of Roc-A (Figure 4). Results for overlap similarity between each selected native ligands and Roc-A, as well as the resolution of each PDB, are shown in Table 2. The values for overlap similarity were determined considering the following contributions: 100% steric (100ste), 100% electrostatic (100elt), 60% steric/40% electrostatic (60ste/40elt), 40% steric/60% electrostatic (40ste/60elt) and 50% steric/50% electrostatic (50ste/elt).

**TABLE 2:** Overlap similarity values between Roc-A and native ligands of complexes analyzed.

PDB ID	Ligand name	Overlap^[a]^					Resolution (Å)^[b]^
		100ste	100elt	60ste/40elt	40ste/60elt	50ste/elt	
2CGX	3D3	0.613006	0.546267	0.538365	0.538086	0.537710	2.20
2BRN	DF1	0.722827	0.392307	0.490008	0.412153	0.447358	2.80
2BRO	DF2	0.707922	0.463492	0.543004	0.475574	0.508122	2.20
2BRM	DFZ	0.783006	0.357218	0.491289	0.359335	0.423177	2.20
2WMU	ZYU	0.629184	0.350906	0.457512	0.417874	0.437330	2.60

100ste = 100% of steric contribution; 100elt = 100% of electrostatic contribution; 60ste/40elt = 60% steric and 40% electrostatic; 40ste/60elt = 40% steric and 60% electrostatic; and 50ste/50elt = 50% of both contributions. ^[a]^: Overlap similarities values obtained using the software Biovia Discovery Studio Visualizer. ^[b]^: Data retrieved from rcsb.org.

The ligand 3D3 presented the highest values of overlap similarity with Roc-A, i.e., 0.546267, 0.538086, and 0.537710 for the contributions 100elt, 40ste/60elt, and 50ste/elt, respectively. Also, the second highest value for the contribution 60ste/40elt (0.538365) and a good value for 100ste (0.613006). Worth reminding that values of overlap similarities closer to 1.0 indicate a greater degree of similarity between Roc-A and the given ligand (Biovia et al., 2000).

**FIGURE 5:**
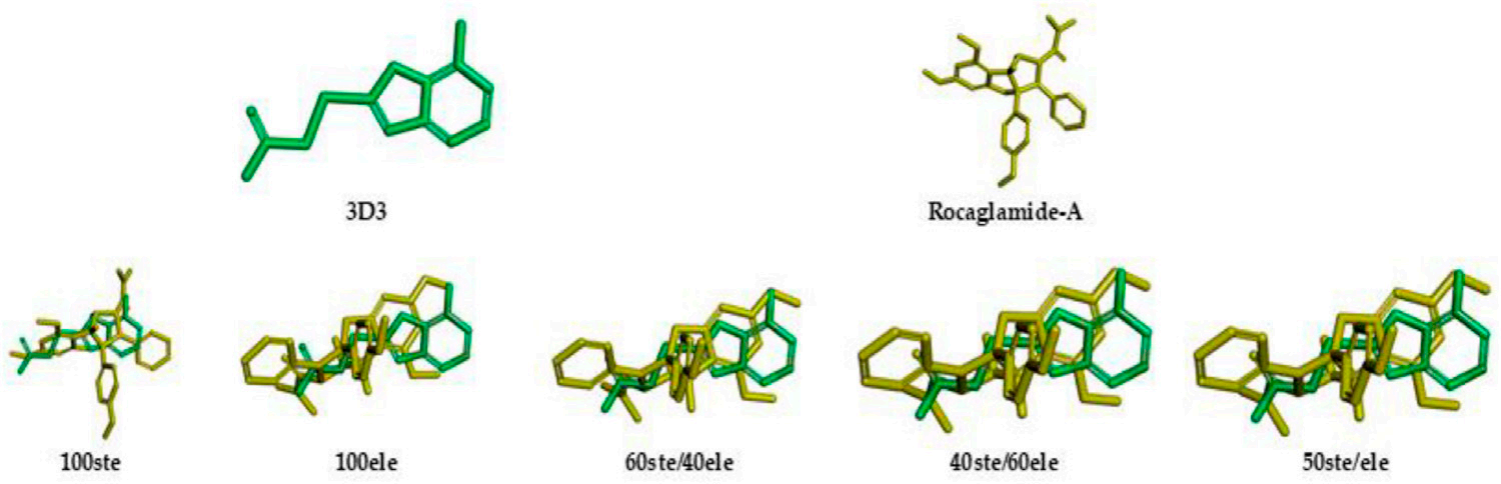
Representations of overlap similarities between molecular structures of 3D3 ligand (green) and Rocaglamide-A (yellow) according to different steric/electrostatic contributions.

These results allowed us to assume a reasonable degree of similarity between the ligand 3D3 and Roc-A. Figure 5 shows the ligand 3D3 (IUPAC name 2-[(6-amino-7H-purin-8-yl)thio]acetamide) overlapped with Roc-A. Such ligand complexed with the protein Chk1, was deposited by Foloppe et al. (2006) under the PDB code 2CGX. Moreover, this protein/complex crystallographic structure was chosen because it presents a resolution of 2.20 Å, which indicates its suitability for molecular docking studies.

### Docking Studies for the Training Set

After selecting the Chk1 protein complex (PDB ID 2CGX), we performed docking simulations using the GOLD software, following the procedures detailed in the methodology section (topic *Docking Studies for the Training Set*). Before running docking simulations for the 15 compounds of the training set, validation of the docking protocol in the GOLD software was performed. The validation procedure was successful, as can be seen in Supplementary Figures S1, S2 (see Supplementary Material). The native 3D3 ligand was redocked into the Chk1 protein showing an RMSD of 1.15 Å and keeping the same relevant interactions within the binding site.

In sequence, we ran docking simulations for Roc-A and retrieved its best scored pose by GOLD. Such pose can be depicted by two main key interactions: hydrogen bond between the OMe and NH group of Cys87, and a hydrogen bond between the OH and C=O of Leu15 (see Supplementary Figure S2). When docking the other 14 compounds from the training set, these same two interactions (along with others) were observed (Supplementary Figure S2). Regarding the score values for compounds, Roc-A showed a value of 38.873. It is noteworthy that this value was lower than those observed for all other 14 compounds in the training set (shown in Table 3), which suggests that the whole training set is suitable for building pharmacophore models.

**TABLE 3:** Compounds from the training set ranked according to their corresponding docking score values, predicted using the CHEMPLP scoring function by GOLD software.

Compound	Score (GOLD—CHEMPLP)
Roc-A	38.873
7	51.520
5	50.822
2	49.503
11	49.045
3	48.018
15	47.828
14	46.745
9	46.104
4	45.177
8	45.146
10	44.818
13	43.561
12	42.213
6	41.283

### Pharmacophore Modeling

**FIGURE 6:**
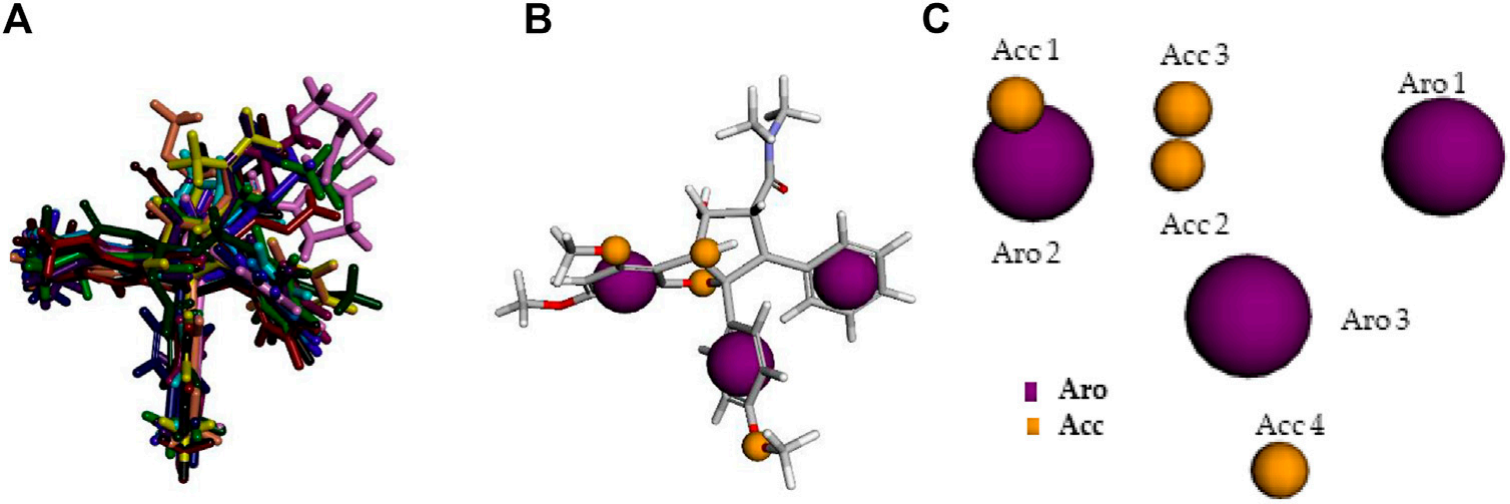
Qualitative characteristics of the best pharmacophore model initially generated by PharmaGist. **(A)** Aligned molecules. **(B)** Pharmacophoric features positioned over Roc-A (pivot molecule). **(C)** Pharmacophoric features: 3 aromatic groups (Aro) in purple, and 4 hydrogen bond acceptor groups (Acc) in yellow.

The best pharmacophore model was chosen according to the set of pharmacophoric features presenting highest scores, as well as to the multiple alignment of Roc-A and 14 derivatives. In other words, the Pharmagist webserver generates scored sets of pharmacophoric features based on the alignment of the molecules with the pivot molecule (which is kept rigid). The webserver’s algorithm uses standard weighted values for each pharmacophoric feature. Initially, the alignment of each pivot molecule pair is scored by its characteristics and then the multiple alignment between the best analyzed pairs is generated. Several multiple alignments are, therefore, scored in the same way (Schneidman-Duhovny et al., 2008a; Schneidman-Duhovny et al., 2008b; da Silva Costa et al., 2018). In this manner, the quantitative characteristics for the best pharmacophore model are shown in Table 4 and its qualitative characteristics are shown in Figure 6.

**TABLE 4:** Score, pharmacophoric features, and aligned compounds in the best pharmacophore model generated by PharmaGist.

Score	F	SF	Aro	Hyd	Don	Acc	Neg	Pos	Aligned compounds
64.640	7	7	3	0	0	4	0	0	1*, 2, 3, 4, 5, 6, 7, 8, 9, 10, 11, 12, 13, 14, 15

F, number of features; SF, spatial features; Aro, aromatic groups; Hyd, hydrophobic groups; Don, donor groups; Acc, acceptor groups; Neg, anionic atoms; Pos, cationic atoms. 1*, pivot molecule (Roc-A).

The best pharmacophore model showed a score of 64.640 with all of the fifteen molecules from the training set aligned. Furthermore, it presented seven features (F), that is: 7 spatial features (SF) related to the conformation of pharmacophoric regions; 3 aromatic regions (Aro); and 4 hydrogen bond acceptor groups (Acc). Worth mentioning that the model did not show features regarding hydrophobic regions (Hyd); hydrogen donors (Don); anionic atoms (Neg), and cationic atoms (Pos).

### Evaluation of Pharmacophore Models

In order to evaluate this initial pharmacophore model and to prove the correct alignment of corresponding structures, we carried out an analyses of *p* values and HCA. Table 5 shows the data obtained from this pharmacophore model, for each compound of the training set, as well as corresponding TI calculated values. In addition, it shows *p* values in between pharmacophoric features and TI values.

**TABLE 5:** Pharmacophoric features for each compound of the training set and their TI (Tanimoto Index) similarity values in relation to Roc-A (pivot molecule). Additional matrix showing pearson’s correlation *(p)* values in between pharmacophoric features and TI values.

Compound	F	SF	Aro	Acc	TI
Roc-A	17	15	3	7	1.000000
2	20	17	3	8	0.946588
3	18	16	3	8	0.852853
4	20	17	3	9	0.816619
5	18	17	3	9	0.811429
6	20	18	3	9	0.800562
7	19	17	4	9	0.786885
8	21	17	3	9	0.772989
9	22	18	3	9	0.769886
10	18	17	4	10	0.751958
11	22	20	3	9	0.749311
12	17	15	4	7	0.748588
13	19	18	4	10	0.741026
14	21	20	4	9	0.722222
15	21	19	3	9	0.693431
SF	0.813	**—**	**—**	**—**	**—**
ARO	−0.319	0.000	**—**	**—**	**—**
ACC	0.438	0.624	0.221	**—**	**—**
TI	−0.410	−0.591	−0.422	−0.602	**—**

F, number of features; SF, spatial features; Aro, aromatic groups; Acc, acceptor groups; TI, tanimoto index.

Pharmacophoric features Hyd, Don, Neg, and Pos were not analyzed in terms of *p* values, due to their absence in the respective pharmacophore model. Observation of the *p* values between the pairs of features in the generated matrix, allows one to infer that there was: no Aro-SF correlation (0.000), weak positive Acc-Aro correlation (0.221), moderate positive Acc-F and Acc-SF correlation (0.438 and 0.624), and strong positive SF-F correlation (0.813). Regarding *p* values between TI and pharmacophoric features, only moderate negative correlations were observed (TI-F: 0.410; TI-SF: 0.591; TI-Aro: 0.422, and TI-Acc: 0.602). This is expected, since TI is influenced by the number of bits between two compounds.

The HCA is a complementary multivariate statistical technique widely accepted in the analysis of experimental data (Macêdo et al., 2015; Ferreira et al., 2019). This statistical method was here used in order to select the pharmacophoric features overall correlated with similarity (TI values).

**FIGURE 7:**
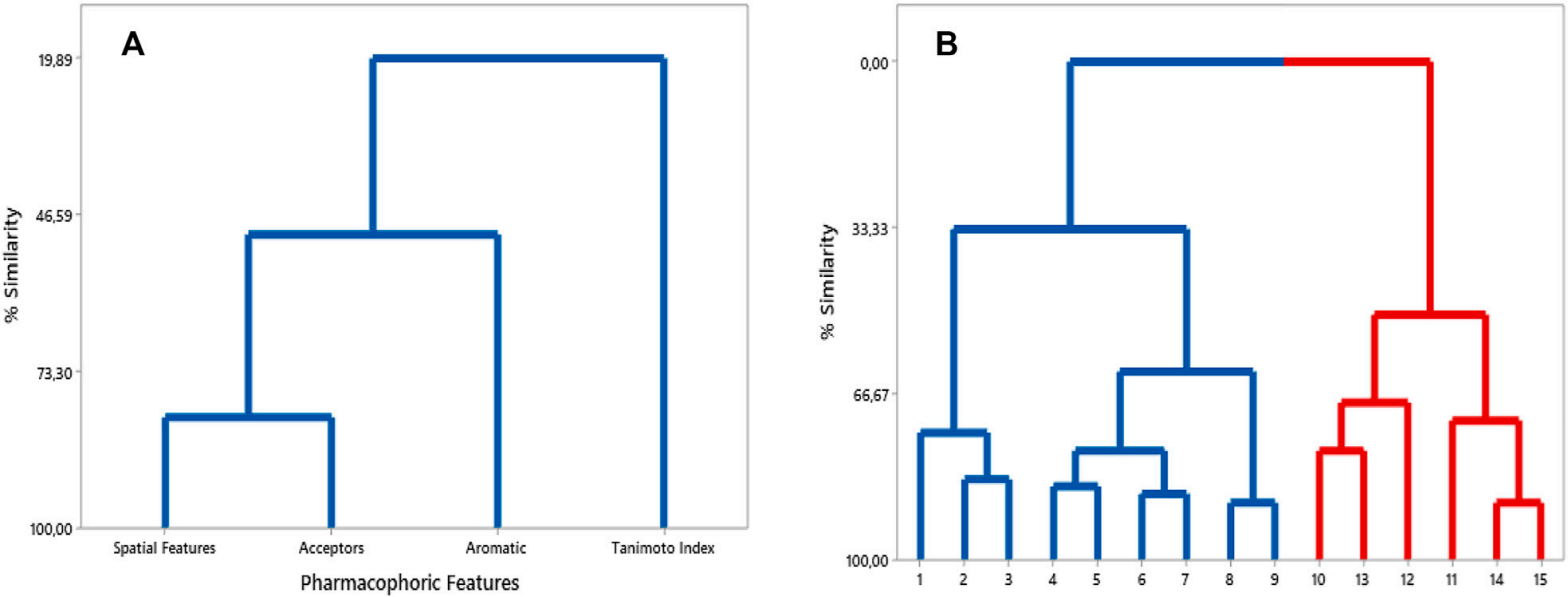
**(A)** HCA dendrogram built considering pharmacophoric features and TI values. **(B)** HCA dendrogram for compounds of training set - more similar in blue cluster and less similar in red cluster.

We constructed an HCA dendrogram which furnished similar results to those of *p* values analysis. Worth noting that the Euclidean distance was used as a parameter to organize the variables into clusters, and also that the pharmacophoric features were considered as dependent variables. In this way, according to this dendrogram, the following correlations could be confirmed: SF, Acc, Aro and TI. That is: these pharmacophoric features remained organized into a single cluster, in which a greater similarity was observed for SF and Acc, followed by Aro and TI (Figure 7A).

Moreover, another HCA dendrogram was built considering the compounds from the training set as observations. This classified them into two clusters: one containing the eight most similar compounds in relation to Roc-A (in blue); and other containing the six least similar ones (in red) (see Figure 7B).

The most similar compounds to Roc-A were molecules 2 and 3. These presented the highest TI values, the same number of Aro, and close numbers of SF and Acc (Table 5). In addition, the overlap similarity, considering 100ste/100ele, between the corresponding pairs between these molecules were: Roc-A–2 (0.829871/0.753159), Roc-A–3 (0.838816/0.713790), and 2–3 (0.981833/0.884395). Also, worth to highlight the overlap similarities for other pairs of molecules, such as: 4–5 (0.925622/0.819664), 6–7 (0.926376/0.866807), and 8–9 (0.945688/0.891420) (Supplementary Table S1). Therefore, these quantitative results of overlap similarity corroborate the alignment of the HCA dendrogram.

**FIGURE 8:**
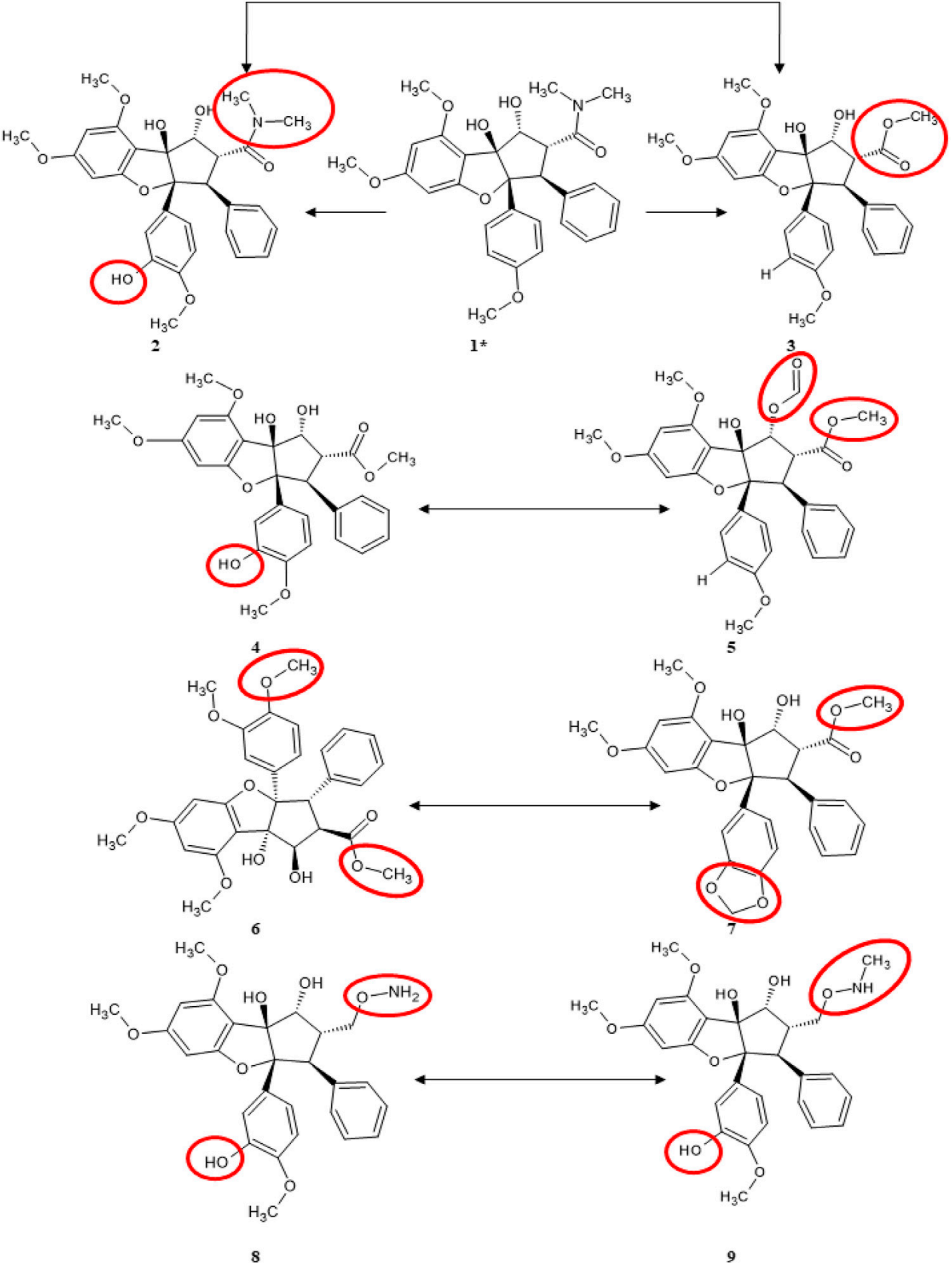
Structural differences between most similar compounds of training set. The red circles indicate which functional groups are exchanged. 1* corresponds to Roc-A (pivot molecule).

We also pointed out structural differences between the molecules of the training set, as can be seen in Figure 8 (which can be conjunctly viewed with Figure 6 that highlights their alignment with the pivot molecule). Worth mentioning some pair’s differences: Roc-A–2 differs by the presence of a hydroxyl group; Roc-A–3 differs by the presence of an ethyl methanoate group; 4–5 differs by the presence of a hydroxyl group in 4, and an ethanal group in 5; 6–7 differs by the presence of a methoxy group in 6, and a 2H-1,3-dioxole group in 7; 8–9 differs by the presence of a methoxylamine group in 8, and an N-hydroxymethanamine group in 9.

### Pharmacophore-based Virtual Screening

The initial pharmacophore model was submitted to the Pharmit webserver (Sunseri and Koes, 2016), to obtain its corresponding spatial coordinates. A set of coordinates was obtained for each of the pharmacophoric features (3 Aro and 4 Acc), which were obtained from aligned molecules, as shown in Table 6. This pharmacophore was here denoted as Model 1 and it was submitted to virtual screening, using the Molport^®^ database, which retrieved only 8 compounds without the application of filters.

**TABLE 6:** Pharmacophoric features and spatial coordinates for pharmacophore Models 1 and 2, obtained by Pharmagist and Pharmit, as well as number of compounds retrieved from corresponding virtual screening campaigns using Molport database.

Model 1^[a]^/Model 2^[b]^					
Pharmacophoric features	Spatial coordinates				Number of compounds obtained
	x	y	z	R	
Acc 1	12.380	−15.578	2.204	0.5	8^[a]^/2^[b]^
Acc 2	15.069	−16.653	−1.457	0.5	
Acc 3	15.205	−15.655	2.057	0.5	
Acc 4	16.838	−21.912	1.729	0.5	
Aro 1	19.831	−16.492	−1.023	1.1	
Aro 2	12.488	−16.602	−0.420	1.1	
Aro 3	16.316	−19.305	0.830	1.1	
				**Total**	**10**

[a] No filters applied.

[b] Filters applied.

Aiming to increase the diversity along the search for new structures, maximum and minimum values of physicochemical properties of the structures were also used as filters (Supplementary Table S2). In this way, we performed another virtual screening in Pharmit considering the Model 2 (with application of filter), which retrieved 2 more compounds. These 2 models, so far, could retrieve a total of 10 compounds (see Table 6) and the graphic spatial distribution of their pharmacophoric features can be seen in Figure 6C.

We realized that Models 1 and 2 were not satisfactory for the virtual screening process, but their pharmacophore alignments were maintained and supported by the evaluation of *p* values, which confirmed the existence of correlation between the variables selected (Figures 6B,C). Moreover, the alignment of structures in more or less similar clusters was also confirmed by the HCA.

So, in order to increase the number of compounds retrieved from virtual screening, as well as their structural diversities, we followed the protocol by (Ferreira et al., 2019) to perform different combinations between pharmacophoric features to generate new pharmacophore models. This has been done by using Eq. 2 (Santos, 2017), presented below:

$$\text{C}p,n=\frac{n!}{p!\left(n-p\right)!}$$ (2)

Where: C = number of combinations; p = model type (*p* ≠ 0, *p* = 1, *p* = 2, *p* = ∞);  n = number of model variables.

Considering a total of 5 variables (pharmacophoric features), by simple combination and without repetition, five new pharmacophore models (Models 3, 4, 5, 6, and 7) were generated. Table 7 details their corresponding set of pharmacophoric features as well as their spatial coordinates. Thus, each one of these new models were further submitted to new virtual screening campaigns, which retrieved us a total of 2.332 compounds—totaling 2.342 compounds out of the seven pharmacophore models.

**TABLE 7:** Pharmacophore Models 3, 4, 5, 6, 7, and corresponding graphic representations, pharmacophoric features, spatial coordinates and number of compounds retrieved in each virtual screening campaign using Molport database.

Graphic representation	Pharmacophoric features	Spatial coordinates				Number of compounds obtained
		x	y	z	R	
**Model 3**						
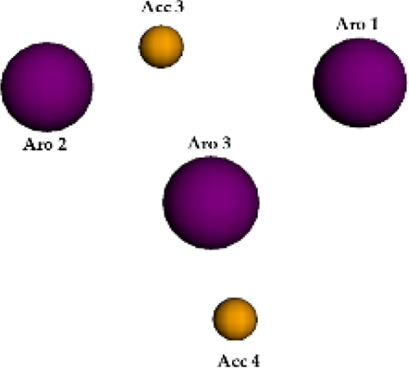	Acc 3	15.205	−15.655	2.057	0.5	991
	Acc 4	16.838	−21.912	1.729	0.5	
	Aro 1	19.831	−16.492	−1.023	1.1	
	Aro 2	12.488	−16.602	−0.420	1.1	
	Aro 3	16.316	−19.305	0.830	1.1	
**Model 4**						
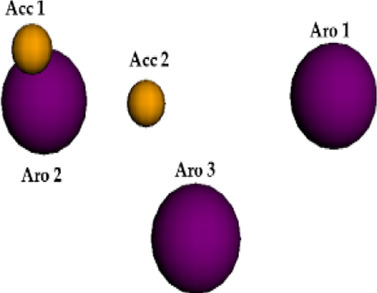	Acc 1	12.380	−15.578	2.204	0.5	129
	Acc 2	15.069	−16.653	−1.457	0.5	
	Aro 1	19.831	−16.492	−1.023	1.1	
	Aro 2	12.488	−16.602	−0.420	1.1	
	Aro 3	16.316	−19.305	0.830	1.1	
**Model 5**						
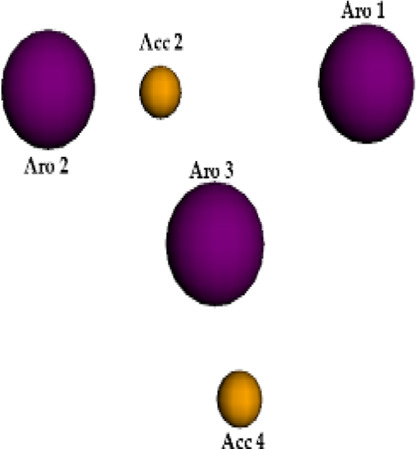	Acc 2	15.069	−16.653	−1.457	0.5	264
	Acc 4	16.838	−21.912	1.729	0.5	
	Aro 1	19.831	−16.492	−1.023	1.1	
	Aro 2	12.488	−16.602	−0.420	1.1	
	Aro 3	16.316	−19.305	0.830	1.1	
**Model 6**						
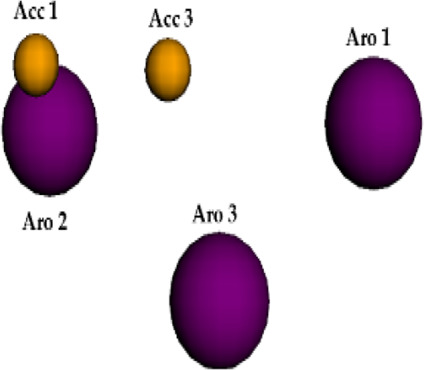	Acc 1	12.380	−15.578	2.204	0.5	217
	Acc 3	15.205	−15.655	2.057	0.5	
	Aro 1	19.831	−16.492	−1.023	1.1	
	Aro 2	12.488	−16.602	−0.420	1.1	
	Aro 3	16.316	−19.305	0.830	1.1	
**Model 7**						
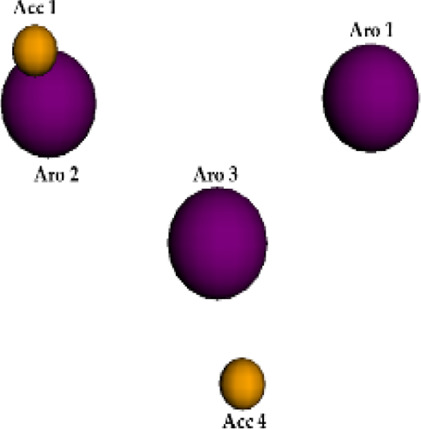	Acc 1	12.380	−15.578	2.204	0.5	731
	Acc 4	16.838	−21.912	1.729	0.5	
	Aro 1	19.831	−16.492	−1.023	1.1	
	Aro 2	12.488	−16.602	−0.420	1.1	
	Aro 3	16.316	−19.305	0.830	1.1	
	**Total**					**2332**

### Selection of Compounds From Pharmacophore-based Screening

In this step, each set of compounds that was obtained from pharmacophore-based virtual screening, employing each model (Models 1, 2, 3, 4, 5, 6 and 7), was considered to calculate corresponding similarities (TI values) in relation to Roc-A. For each set, we sorted similarity values of compounds and selected top 200. Worth reminding that we considered a threshold to only pick up compounds that presented TI value greater than 0.2 (see Table 8). Therefore, we could obtain a total of 931 compounds out of this task, that proceeded to the next screening step.

**TABLE 8:** Number of compounds selected from each pharmacophore model in different thresholds of similarity values (Tanimoto index) in relation to Roc-A.

Tanimoto index thresholds				
≥0.20	≥0.25	≥0.3	≥0.35	≥0.40
**Model 1**				
8	8	8	6	6
**Model 2**				
2	2	2	2	2
**Model 3**				
991	986	814	242	45
**Model 4**				
129	103	74	28	13
**Model 5**				
264	232	150	107	31
**Model 6**				
216	191	116	32	10
**Model 7**				
730	725	562	205	34

### Prediction of Toxicity and Pharmacokinetic Properties

The retrieved 931 compounds, in addition to Roc-A, were subjected to predictions of toxicity and pharmacokinetic properties, as mentioned in methods. This analysis was carried out to filter out and select most promising compounds throughout our screening. In brief, we could retrieve a total of 60 compounds out of these analyses, and Table 9 shows predictions for 10 selected and most relevant compounds - complete pharmacokinetic data on Supplementary Tables S3–S7 (see Supplementary Material). Next, we discuss some important remarks considering our analysis of toxicity and pharmacokinetic predictions.

**TABLE 9:** Prediction of toxicity and pharmacokinetic properties for 10 selected compounds, out of total of 60 retrieved from virtual screening, using DEREK and SwissADME, respectively.

Compound	GIA	BBBP	Lipinski violations	Toxicity endpoint^a^	Toxicophoric group	Toxicity alert
Roc-A	High	No	1	Skin sensitisation	Substituted phenol or precursor	Plausible
				Teratogenicity	4-hydroxydiphenyl-ethane or -ethene	Plausible
**Model 4**						
PC-53093220	High	No	0	—	—	No alert
PC-53116405	High	No	0	—	—	No alert
**Model 5**						
PC-16811025	High	No	0	—	—	No alert
PC-135638768	High	No	0	—	—	No alert
PC-16803784	High	No	0	—	—	No alert
PC-16810169	High	No	0	Skin sensitization	Substituted phenol or precursor	Plausible
PC-18582767	High	No	0	—	—	No alert
PC-16810171	High	No	0	Skin sensitization	Substituted phenol or precursor	Plausible
**Model 6**						
PC-9115580	High	No	0	—	—	No alert
**Model 7**						
PC-17581023	High	No	0	Skin sensitization	Substituted phenol or precursor	Plausible

PC, PubChem; GIA, Gastrointestinal Absorption; BBBP, Blood-Brain Barrier Permeant.

a In human, mouse and/or rat.

Lipinski’s Rule of Five is intended to help medicinal chemists filtering potential drug candidates, by excluding those with unwanted physicochemical properties. According to Lipinski’s Rule of Five, four molecular properties are overall considered: *logP* (≤5) (predicted here as iLOGP), number of hydrogen bond donors (≤5), number of hydrogen bond acceptors (≤10) and molecular weight (<500 g/mol). Furthermore, several extensions of the Lipinski’s rule have been proposed as guidelines and one of them mentions, for instance, that TPSA must be less than 140 Å^2^ (Lipinski et al., 2001; Veber et al., 2002). Considering these parameters, we evaluated that Roc-A presented 1 violation of Lipinski’s rule, since it presents MW = 505.56 g/mol. Nevertheless, all other selected compounds did not present violations of Lipinski’s rule (see Table 9).

GIA and BBB permeation are two crucial pharmacokinetic characteristics for developing drug candidates. Although there are different routes of drug administration, the oral route is generally preferred due to patient’s comfort. Thus, initial estimation of oral bioavailability, that is, the fraction of the dose that reaches the bloodstream after oral administration, is a key decision-making criterion at various stages of the drug development process. Worth noting that bioavailability is highly multifactorial, but is primarily driven by GIA (Newby et al., 2015). BBB may be considered a shield that protects the brain, since it is a “physical” and “biochemical” barrier. Although active transport is important, passive diffusion is the main pathway for drugs to access the brain from the bloodstream (Di et al., 2012). Therefore, for a drug with biological activity in the central nervous system (CNS), a favorable BBB permeation is desirable. However, for a drug with no CNS activity, as drawn here, permeation to the BBB is not necessary, so that side effects are minimized (Wang and Hou, 2009; Rojas et al., 2011). GIA and BBB permeation were here predicted by the BOILED-Egg model proposed by Daina and Zoete, (2016). Such model outputs “high” or “low” for GIA, and “yes” or “no” for BBB permeation. Our predictions showed that Roc-A and all other compounds presented “high” GIA and “no” BBB permeation (see Table 9), a fact that makes them suitable for the next stages of the study.

Toxicity predictions were carried out to verify and investigate toxicity alerts, such as the presence of toxic groups (toxicophoric) in the compounds. From Table 9, we observe that Roc-A showed skin sensitization toxicity alert, which refers to allergic response produced by contact of a substance with the skin (Aptula et al., 2005). This alert has been attributed as plausible due to the toxicophoric group substituted phenol or precursor. In addition, there was also a toxicity alert for teratogenicity, which refers to the possibility of a substance causing fetal malformation during the gestational period. This alert was attributed as plausible to the toxicophoric group 4-hydroxydiphenyl-ethane or -ethene. From the remaining compounds, 41 of them showed no toxicity alerts, but 24 of them showed plausible toxicity alerts for skin sensitization. Among the toxicophoric groups for the latter are: substituted phenol or precursor, phenyl ester, activated N-heterocycle, hydrazine or precursor, imine or alpha, beta-unsaturated imine.

To sum up, Roc-A showed a toxicity alert for teratogenicity and one violation of Lipinski’s rule, while the screened compounds did not show similar alerts. These results allowed us to infer that, overall, the screened compounds presented improved toxicity and pharmacokinetic predicted profiles than the pivot molecule (Roc-A).

### Docking-Based Virtual Screening

We carried out docking simulations using three different software, following procedures detailed in methodologies section. The idea of using three software—GOLD, FRED and Dockthor, which employ different methodologies—was to expand and diversify possible interpretations for ligand-protein interactions as well as to analyze their corresponding scores punctuations in a consensual perspective.

In advance of running docking simulations to the 60 remaining compounds, validation of each docking protocol in 3 software were performed. Validation procedures were all successful in three cases as one can see in Figure S1. The native ligand 3D3 was redocked into Chk1 protein presenting a RMSD of 1.15, 1.53 and 1.64 Å by using the software GOLD, FRED and Dockthor, respectively.

**FIGURE 9:**
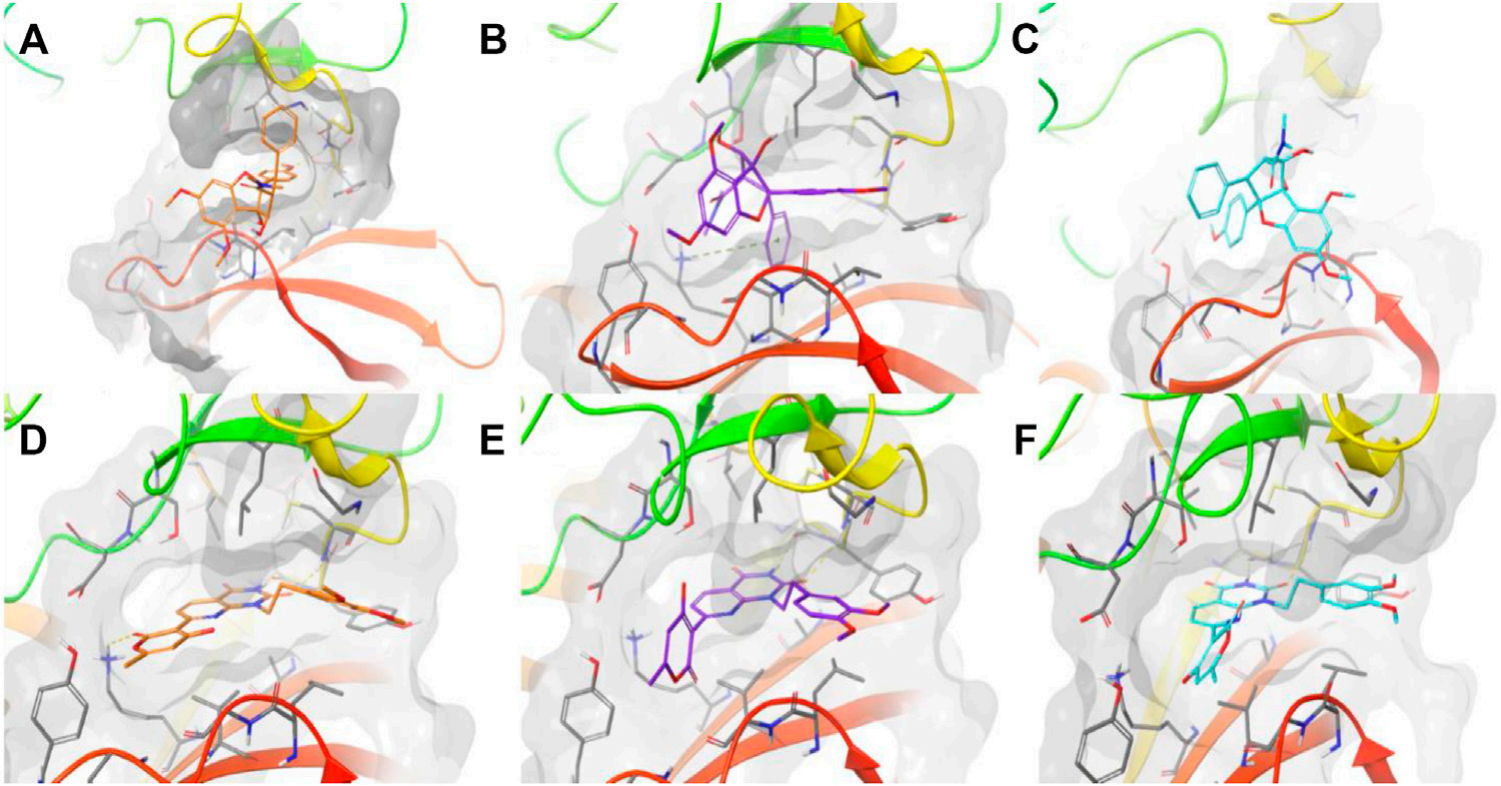
Docking poses obtained for Roc-A using **(A)** GOLD in orange, **(B)** FRED in purple, and **(C)** Dockthor in light blue, and for PubChem-135638768 **(D,E,F)** using same corresponding software/colors. Results obtained using the protein Chk1 (PDB ID 2CGX). Dashed lines in yellow represent hydrogen bonds and in green cation-pi. Figures were prepared using Maestro.

Moreover, we ran docking simulations to Roc-A using the three software. Figure 9 shows the obtained docking poses for the pivotal compound. One should note that poses were not too similar regarding their overlaps and distribution within binding site, moreover different interactions were observed: GOLD pose showed one hydrogen bond between OMe group and NH from Cys87 and one hydrogen bond between OH and C=O from Leu15; FRED pose showed one pi-cation interaction between phenyl and ^+^NH_3_ from Lys38; and Dockthor pose showed no interactions, to view the interactions in more detail see Supplementary Figure S3. Score values obtained for Roc-A was 38.873, −4.889, and −7.816 using GOLD, FRED and Dockthor, respectively. Worth mentioning that these values were worse than those observed for the majority of 60 screening compounds, in general, which could suggest that this set of screening compounds show an even greater potential to interact with Chk1 than Roc-A.

**FIGURE 10:**
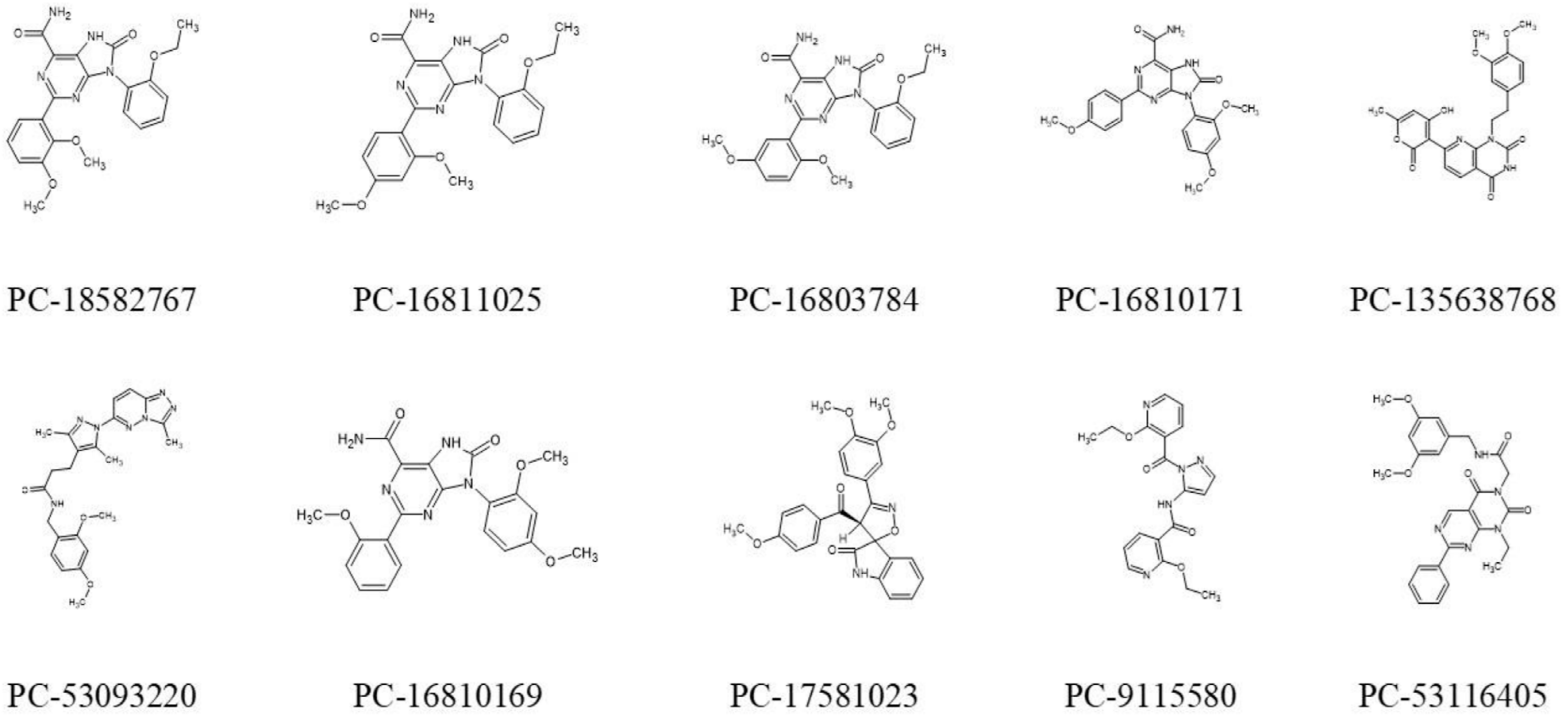
Representation of 2D structures of 10 promising compounds obtained by hierarchical virtual screening. PC: PubChem.

Docking results for the 60 screening compounds showed that each one of the 3 software was able to generate diverse poses for them. However, our consensus analysis was aimed at considering corresponding scores obtained. In other words, we performed consensus scoring with the docking results. In this way, each table output, from each software (containing the score values for each compound), were sorted in ranking values from best to worst (1–60 position). These ranking values were then considered to calculate an average ranking value for each compound. Thus, we were able to pick top 10 compounds with best average ranking values obtained by using 3 docking software, as shown in Table 10 and their 2D structures in Figure 10.

**TABLE 10:** Top 10 compounds selected according to their best average ranking values, which in turn were calculated from each individual ranking and corresponding scores obtained using 3 docking software (GOLD, FRED and Dockthor).

Compound	GOLD		FRED		Dockthor		#Average ranking
	score^a^	# Ranking	score^b^	# Ranking	score^c^	# Ranking	
Roc-A	38.8725	37	−4.8893	36	−7,816	31	**34,7**
PC-135638768	71.8748	2	−10.2612	1	−8.998	3	**2.0**
PC-18582767	64.5324	6	−9.8941	2	−8.640	9	**5.7**
PC-53093220	67.2732	3	−8.0923	14	−9.127	2	**6.3**
PC-16803784	65.4361	5	−8.9784	8	−8.633	10	**7.7**
PC-16811025	62.8643	9	−9.7540	4	−8.534	14	**9.0**
PC-16810171	63.3645	8	−9.8666	3	-8.342	19	**10.0**
PC-16810169	62.3539	10	−9.1083	7	−8.502	16	**11.0**
PC-53116405	77.7655	1	−6.9509	29	-−8.734	6	**12.0**
PC-17581023	58.6058	17	−8.1517	12	−8.659	8	**12.3**
PC-9115580	61.9378	12	−7.8324	18	−8.722	7	**12.3**

a GOLD, score values obtained by CHEMPLP, scoring function.

b FRED, Chemgauss4 score values.

c predicted binding affinity by the DockTScore program given in kcal/mol units. PC: PubChem.

In addition, docking poses are presented in Figure 9 for compound PC-135638768 which was considered the top 1 compound in our consensus docking analysis. Interestingly, the three poses obtained by three software showed a great overlap and the same hydrogen bond interactions between NH and C=O from Glu85 and C=O and NH from Cys87. Additionally, GOLD pose presented a hydrogen bond between C=O and ^+^NH_3_ from Lys38. One should note that this latter interaction was verified in a similar way as for Roc-A FRED pose. Also, the crucial interaction observed for the native ligand with Cys87 has been overall kept for compound PC-135638768. Therefore, this suggests that the final top 10 compounds selected by these criteria may show a great potential to act as putative Chk1 inhibitors with interest in anti-skin cancer activity.

### Analysis of Properties and Structures of Promising Compounds

The *1*-octanol/water partition coefficient *logP* is commonly used as a parameter to express a given compound’s lipophilicity, which is a key property for drug development (Daina et al., 2014). Such property affects, for instance, the tendency of a compound to break down into non-polar versus aqueous environments. Therefore, increasing the lipophilicity of compounds, generally, might lead to increase on their permeability, protein binding, volume of distribution, as well as decrease on their solubility and renal excretion (Kerns and Di, 2003).

**FIGURE 11:**
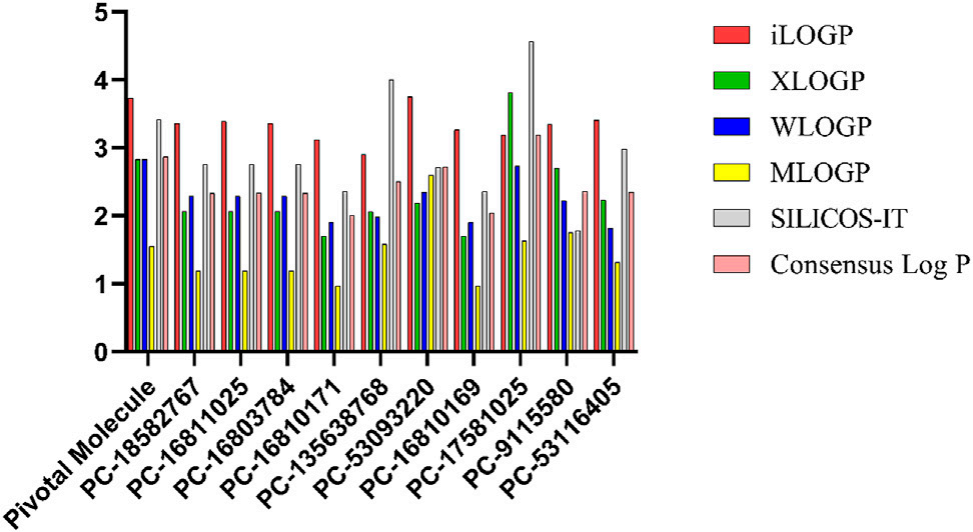
*logP* values predicted using different methodologies for pivotal molecule and 10 promising compounds. Pivotal molecule: Roc-A; PC: PubChem.

Roc-A presented a consensus *logP* value equal to 2.87; while the 10 promising compounds showed consensus *logP* values spanning from 2.01 to 3.19, as can be seen in Figure 11 and Supplementary Tables S8–S13. In fact, in this study, only positive *logP* values in the range of 0.97–4.57 were found. Worth mentioning that such positive values indicate reasonable lipophilicities, according to Sepay et al. (2020). Compounds PC-18582767, PC-16811025 and PC-16803784 have similar chemical structures, bearing an ethoxyphenyl group and differing only in the positions of their dimethoxyphenyl groups, which attributes them similar *logP* values. On the other hand, PC-9115580 and PC-53116405 do not bear an ethoxyphenyl group, so the insignificant change in their *logP* values indicates that the absence of this group cannot increase their polarity. In addition, PC-16810171 and PC-16810169 are chemically similar and differ only in the position of their methoxyphenyl groups, so they have very similar *logP* values. Lastly, PC-135638768 has 3 nitrogen atoms in its structure and a methyl group, which may explain its higher *logP* value, favoring solubilization in a hydrophobic medium.

According to Sepay et al. (2020) water solubility is also an important requirement for any drug candidate intended to be administered orally, or parenterally, since a sufficient amount of active pharmaceutical ingredients must be administered in a small volume.

**FIGURE 12:**
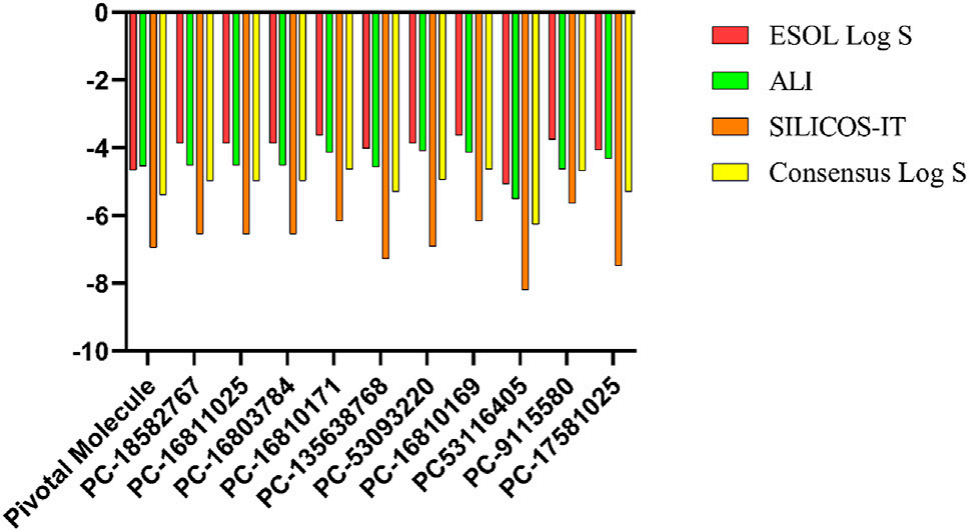
*logS* values predicted using different methodologies for pivotal molecule and 10 promising compounds. Pivotal molecule: Roc-A; PC: PubChem.

Roc-A presented a consensus *logS* value equal to -5.38; while the promising compounds showed consensus *logS* values in the range of −4.64 to −6.26, as shown in Figure 12 and Supplementary Table S14. In this study, only negative *logS* values in the range −3.63 to −8.20 were found. According to Sepay et al., 2020, *logS* values between −4 and −6 indicate moderate solubility, −2 to −4 indicate good solubility, and greater than -6 indicate poor solubility. Therefore, we infer that Roc-A and nine promising compounds were moderately soluble in water, and PC-17581023 is poorly soluble in water. This suggests that the majority of promising molecules found here might be administered orally.

Additionally, we searched for our 10 promising compounds in the Scifinder^®^ and found no biological activity or patent previously reported for them, which make them novel chemical structures in this context.

Furthermore, in order to corroborate the hierarchical virtual screening data and verify the similarity between the promising compounds and Roc-A, we carried out a similarity analysis taking into account the overlap of their steric fields. Steric factors represent a fundamental characteristic related to the shape and conformation of chemical structures, being commonly associated with their potential biological activity (McConathy and Owens, 2003). From Table 11, one can see that the 10 promising compounds showed overlap similarities, in relation to Roc-A, ranging from 35 to 56% for 50ste, from 46 to 60% for 70ste, and from 57 to 69% for 100ste. Also, Supplementary Figure S4 shows the overlap poses between Roc-A and the promising compounds. Compound PC-18582767 presented the highest overlap similarity in 50ste, and PC-16803784 presented highest overlap in both 70ste and 100ste.

**TABLE 11:** Overlap similarity values of the 10 promising compounds in relation to Roc-A.

Compound	Overlap		
	50ste/elt	70est/30elt	100ste
PC-135638768	0.482649	0.514834	0.601549
PC-18582767	0.566805	0.574828	0.668316
PC-53093220	0.452489	0.479363	0.570985
PC-16803784	0.556497	0.607117	0.698289
PC-16811025	0.472699	0.512293	0.681568
PC-16810171	0.418454	0.462070	0.688019
PC-16810169	0.459229	0.543432	0.691514
PC-53116405	0.488060	0.513044	0.616279
PC-17581023	0.357190	0.500264	0.658258
PC-9115580	0.511303	0.547238	0.613247

PC, PubChem; 50ste/elt, 50% of both contributions; 70ste/30elt, 70% steric and 30% electrostatic; 100ste, 100% of steric contribution.

In general, these results allowed us to infer that all 10 promising compounds present reasonable similarity with Roc-A, which is a known compound with bioactivity in experiments using SC cells. Insightfully, also considering the docking results, the 10 compounds retrieved in this work are likely to present potential anti-SC activity.

### *In silico* Evaluation of Selectivity and Theoretical Determination of Biological Activity

In order to validate the molecular docking methodology, the crystallographic ligands were selected for redocking using the AutoDock 4.2/Vina 1.1.2 software, via PyRx graphical interface, with the crystallographic poses of the receptors: Chk1 (PDB ID 2CGX) (Foloppe et al., 2006), elF4A1-ATP (PDB ID 5ZC9) (Iwasaki et al., 2019) BRAF kinase (PDB ID 6XFP) (Yen et al., 2021).

From the crystallographic poses and obtaining the computational poses of the respective inhibitors (3D3, RCG and V1Y) complexed to the proteins, it was possible to perform the validation of the molecular docking methodology by calculating the RMSD between the poses. The results obtained were 1.58, 1.43 and 0.50 Å, respectively. According to Gowthaman et al. (2008) and Hevener et al. (2009), the docking methodology is validated when the RMSD value calculated between the crystallographic and computational poses is less than 2.0 Å. The best results can be seen in Supplementary Figure S5.

Foloppe et al. (2006) reported the discovery of 10 new Chk1 inhibitors, distributed in 9 different chemical structures. All ligands act by competitive binding to the ATP target site. According to crystallographic data deposited by Foloppe et al. (2006), at the site of the Chk1 protein (PDB ID 2CGX), the 3D3 inhibitor complexed performs Pi-Sigma interactions with the Val23 and Leu137 residues and Pi-Alkyl with the Ala36 residue in the *ß*-sheet. Leu15 and Cys587 residues show Pi-Alkyl type interaction in the Loop region of the protein. It is possible to see Conventional Hydrogen Bonding interactions of residues Gly16, Tyr20 and Cys87, and Carbon-Hydrogen Bonding interactions with residue Glu85, all in the Loop region. Residues Lys38 and Gly90 perform Van der Waals interactions in the *ß*-sheet, as well as residues Gly18, Val 68, Glu85, Tyr86 and Asp148 in the Loop.

Iwasaki et al. (2019) reported that Roc-A exhibits antitumor activity by binding eukaryotic initiation factor-4A (eIF4A) to polypurine mRNA sequences. The RCG inhibitor complexed in the protein elF4A1-ATP (PDB ID 5ZC9) performs Pi-Pi T-Shapped interactions with the Phe163 residue, Conventional Hydrogen Bonding with Gln195 residue and Van der Waals interactions with Asp16, Arg110, Pro159, Gly160, Asp198 and Ile199 residues, all in the α-helix. It is important to emphasize the interactions of the inhibitor with nucleotides of the RNA strand present in the protein structure. The G8 nucleotide performs Pi-Alkyl, Pi-Pi T-Shaped and Conventional Hydrogen Bonding interactions, as well as the A7 nucleotide, according to studies conducted by Iwasaki et al. (2019).

The RAFs proteins (ARAF, BRAF and CRAF) are fundamental for the signaling of the RAS-RAF-MEK-ERK (MAPK) pathway, which is central in the regulation of cell growth and proliferation. In addition, half of malignant melanomas contain BRAF mutations (Sekulic et al., 2008). The V1Y complexed inhibitor at the active site of the BRAF receptor (PDB ID 6XFP), in the Loop region, shows Pi-Pi T-Shapped interactions with Trp531 and Phe595 residues, Pi-Alkyl with Ile513, Leu514, His574 and Cys532 residues, and Pi-Sulfur interactions with the Phe595 residue waste Cys532 and Asp594 show interactions of the Conventional Hydrogen Bonding type, and residues Trp531 and Gly593 show interactions of the Carbon-Hydrogen Bonding type. Only residue Leu593 showed Van der Waals interactions in that region. In the α-helix regions, the inhibitor performs Pi-Alkyl interactions with residues Leu505 and Leu567, Conventional Hydrogen Bonding with residue Glu501. In *ß*sheet regions, the inhibitor performs Pi-Alkyl interactions with residues Val471, Ala481 and Lys483, Pi-Cation with residue Lys483, Carbon-Hydrogen Bonding with residue Gln530 and Van der Waals interactions with residues Ile463, Val482, Ile527, Phe583 and Ile592, according to a study developed by Yen et al. (2021).

**FIGURE 13:**
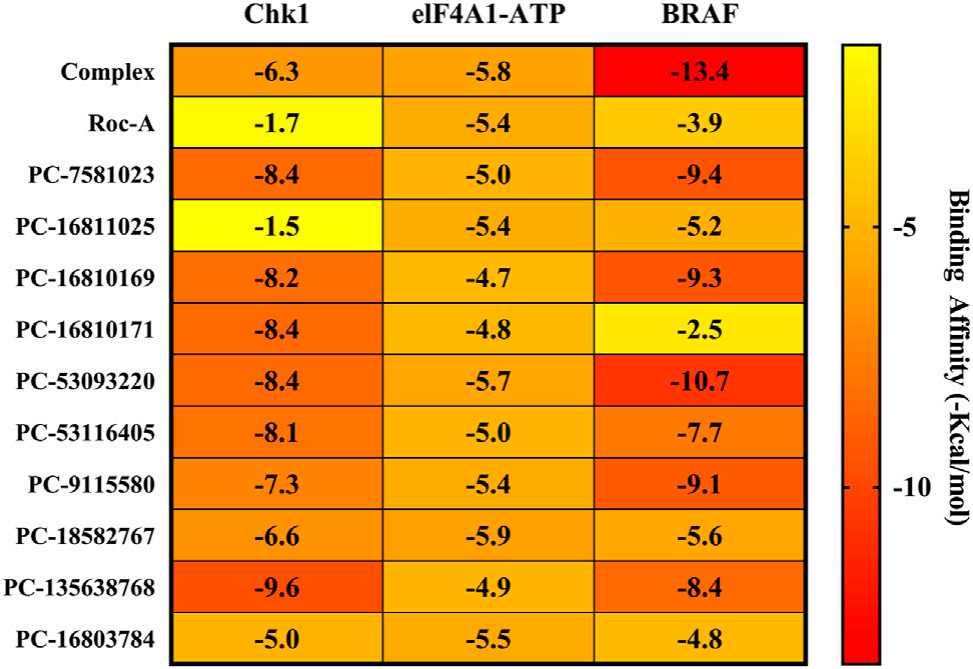
Heatmap plot comparing the experimental, commercial and promissing compounds binding affinity (ΔG) values in receptors: Chk1 (PBD ID 2CGX), elF4A1-ATP (PDB ID 5ZC9) and BRAF (PDB ID 6XFP).

In order to evaluate if promising compounds have binding affinity (∆G) superior to complexed inhibitors (3D3, RCG and V1Y) and to the control compound (Roc-A) at the active sites of Chk1, elF4A1-ATP and BRAF receptors, they were submitted to Molecular Docking. The promising compound that showed significant results at the three receptors were PC-53093220, as we can see in Figure 13.

At the Chk1 receptor (PDB ID 2CGX), the 3D3 inhibitor showed a binding affinity (ΔG) of -6.3 kcal/mol. The control compound Roc-A exhibited a binding affinity of -1.3 kcal/mol. The promising compounds PC-7581023, PC-16810169, PC-16810171, PC-53093220, PC-53116405, PC-9115580, PC-18582767 and PC-135638768 showed higher binding affinity results than the control compound (Roc-A) and to the complexed inhibitor (3D3), as shown in Supplementary Figure S6.

At the active site of the elF4A1-ATP (PBD ID 5ZC9) receptor, the RCG inhibitor showed a binding affinity of -5.8 kcal/mol. The control compound (Roc-A) had a binding affinity of -5.4 kcal/mol. Among the promising compounds, only one showed higher binding affinity to the control compound (Roc-A) and/or complexed inhibitor (RCG). That compound was PC-18582767, which exhibited a binding affinity of -5.9 kcal/mol. The promising compounds PC-53093220 and PC-16903784 showed a binding affinity of -5.7 and -5.5 kcal/mol, respectively, higher than the control compound (Roc-A), as shown in Supplementary Figure S7.

At the BRAF receptor (PDB ID 6XFP), the complexed inhibitor V1Y exhibited a binding affinity of -13.4 kcal/mol, while the control compound Roc-A showed a binding affinity of -3.9 kcal/mol. Except for compound PC-16810171, all other promising compounds showed higher binding affinity than the control compound Roc-A. However, only a few promising compounds approached the binding affinity of the complexed ligand, they are PC-53093220, PC-7581023, PC-16810169 and PC-9115580, exhibiting respectively -10.7, -9.4, -9.3 and -9.1 kcal/mol. In comparative terms, the promising compound that exhibited the highest binding affinity (PC-53093220) has a difference of ±2.03 kcal/mol in relation to the receptor-complexed inhibitor (V1Y), as we can see in Supplementary Figure S8.

In Supplementary Figures 9S–12S (Supplementary Material) are shown the interactions of complexed inhibitors with amino acid residues at the respective active sites (A) 3D3 (PDB ID 2CGX) (B) RCG (PDB ID 5ZC9) and (C) V1Y (PDB ID 6XFP), as well as the interactions of promising compounds [(A) PC-135638768 (B) PC-53093220 and (C) PC-7581023] with amino acid residues in the active site of the Chk1 receptor (PDB ID 2CGX). Furthermore, they are shown the interaction of promising compounds [(A) PC-18582767 (B) PC-16903784 and (C) PC-53093220] with amino acid residues in the active site of the elF4A1-ATP receptor (PDB ID 5ZC9), as well as the interaction of promising compounds [(A) PC-53093220 (B) PC-7581023 and (C) PC-9115580] with amino acid residues in the active site of the BRAF kinase receptor (PDB ID 6XFP).

Roc-A and 10 promising compounds were subjected to biological activity prediction analysis in the online PASS software and those with Pa > Pi were plotted (Table 12). Therefore, Roc-A presented as predicted biological activities related to SC: antineoplastic, antineoplastic (squamous cell carcinoma), apoptosis agonist e kinase inhibitor.

**TABLE 12:** Prediction of biological activity and cytotoxic effect of promising compounds via PASS online and CLC-Pred, respectively.

CLC-PRED^[^ ^a^ ^]^						PASS^[^ ^b^ ^]^		
Compound	Pa	Pi	Cell-line	Cell-line full name	Tissue	Pa	Pi	Activity
Roc-A	0.198	0.139	M19-MEL	Melanoma	Skin	0.920	0.050	Antineoplastic
						0.862	0.001	Antineoplastic (squamous cell carcinoma)
						0.809	0.008	Apoptosis agonist
						0.394	0.069	Kinase inhibitor
PC-135638768	0.129	0.012	SK-MEL	Melanoma	Skin	0.693	0.015	Apoptosis agonist
						0.379	0.112	Antineoplastic
						0.297	0.148	Kinase inhibitor
PC -18582767	0.351	0.038	SK-MEL-5	Melanoma	Skin	0.289	0.159	Antineoplastic
	0.216	0.158	Malme-3M			0.223	0.191	Apoptosis agonist
	0.136	0.123	A-431					
PC-16803784	0.358	0.036	SK-MEL-5	Melanoma	Skin	0.239	0.195	Antineoplastic
	0.216	0.156	Malme-3M					
PC-16811025	0.338	0.042	SK-MEL-5	Melanoma	Skin	0.247	0.188	Antineoplastic
	0.208	0.172	Malme-3M			0.218	0.196	Apoptosis agonist
						0.242	0.232	Kinase inhibitor
PC-16810171	0.270	0.073	SK-MEL-5	Melanoma	Skin	0.302	0.151	Antineoplastic
	0.248	0.109	Malme-3M			0.283	0.166	Kinase inhibitor
						0.221	0.007	Antineoplastic enhancer
PC-16810169	0.413	0.025	SK-MEL-5	Melanoma	Skin	0.289	0.159	Antineoplastic
	0.220	0.150	Malme-3M			0.280	0.170	Kinase inhibitor
						0.174	0.134	Antineoplastic enhancer
PC-17581023	0.236	0.102	M19-MEL	Melanoma	Skin	0.424	0.056	Kinase inhibitor
	0.215	0.159	SK-MEL-28			0.303	0.151	Antineoplastic
	0.198	0.171	M14					
PC-9115580	0.753	0.005	UACC-257	Melanoma	Skin	0.224	0.006	Protein kinase inhibitor
	0.613	0.010	SK-MEL-2					
	0.601	0.008	M14					
	0.569	0.011	LOX-IMVI					
	0.533	0.014	SK-MEL-28					
	0.524	0.014	SK-MEL-5					
	0.372	0.030	UACC-62					

PC, pubchem; Roc-A, Rocaglamide-A

a Cell Line Cytotoxic Predictor.

b Prediction of Activity Spectra for Substances.

The predicted biological activities of the promising compounds were similar to those of Roc-A. However, the promising compounds PC-16811025 and PC-16810169 presented as predicted biological activity related to SC “Antineoplastic enhancer”, which is absent in the prediction of Roc-A (Table 12).

Table 12 also shows prediction results via CLC-Pred (Pa > Pi) of Roc-A and promising compounds under SC cell lines (SCCL). Therefore, Roc-A showed predicted cytotoxic effect against 1 SCCL (M19-MEL). It is worth emphasizing that Roc-A has experimental IC_50_ data in SCCL (RPMI-7951 and kB cells) that corroborate its cytotoxic activity predicted here (Wu et al., 1997).

Most promising compounds had a cytotoxic effect against more than 1 SCCL. In addition, the promising compound PC-9115580 had predicted cytotoxic activity against 8 SCCL. However, 2 promising compounds (PC-53093220 and PC-53116405) did not show predicted biological or cytotoxic activity (Pa > Pi). This fact should not be seen as a lack of activity, since even compounds with known and potent activity can present a low value of Pa or even Pa < Pi during the prediction (Ramos et al., 2022; Lagunin et al., 2018).

## Conclusion

Computer-assisted drug design is currently a reality by which is possible to save time and resources for the treatment of existing diseases, or new ones that may arise, such as SC. Studies conducted here have shown that Roc-A and its derivatives were a good starting point to apply molecular modeling strategies. More specifically, the selected fourteen Roc-A derivatives presented better predicted affinities for the Chk1 receptor than Roc-A itself, suggesting that their use was a good path to develop pharmacophore models and subsequently perform virtual screening.

The initial pharmacophore model that has been built consisted of seven pharmacophoric features and after its successful validation it has been expanded to further pharmacophore models. These were, therefore, employed on independent virtual screening campaigns to obtain potential virtual hits. Afterwards, prediction of toxicity and pharmacodynamic properties allowed us to filter out sixty promising compounds for molecular docking.

Consensus docking has been applied to expand and diversify the possible protein-ligand interactions, as well as to consensually analyze their corresponding scores. In this way, it has been found that most of compounds scored higher than Roc-A and, in addition, this furnished us 10 promising compounds with great potential to interact with Chk1. Furthermore, these have well succeeded on analyses considering their structural properties and similarity with Roc-A.

In short, this study depicts a valuable application of hierarchical virtual screening, involving ligand- and structure-based methodologies to propose new potential anti-SC agents. The suggested 10 promising compounds found here have shown better protein-ligand interactions and lower toxicity when compared to the reference compound Roc-A. Finally, these promising compounds should be selected for *in vitro* and *in vivo* tests, as the results of the prediction of biological and cytotoxic activity against SCCL indicate great potential for their use in the treatment of SC.

## Data Availability

The original contributions presented in the study are included in the article/Supplementary Material, further inquiries can be directed to the corresponding author.
