# Supplementary material for: Hierarchical Virtual Screening Based on Rocaglamide Derivatives to Discover New Potential Anti-Skin Cancer Agents
Source: Front Mol Biosci. 2022 Jun 2;9:836572. doi: 10.3389/fmolb.2022.836572 (PMC9201829; doi:10.3389/fmolb.2022.836572)
Supplement: Supplementary file 1 [file Table1.docx]

| **Table S1:** Similarity analyses for the molecular overlap of the pair of structures for 100ste and 100elt | | |
| --- | --- | --- |
| **Similarity of Overlay(%)** | | |
| **Pair of compounds** | **100ste^[a]^** | **100elt^[b]^** |
| **1*-2** | 0,829871 | 0,753159 |
| **1*-3** | 0,838816 | 0,713790 |
| **2-3** | 0,981833 | 0,884395 |
| **4-5** | 0,925622 | 0,819664 |
| **6-7** | 0,926376 | 0,866807 |
| **8-9** | 0,945688 | 0,891422 |
| ***** Pivot molecule. **^[a]^** 100%ste=100% of the steric contribution of the compounds. **^[b]^** 100%ele=100% of the electrostatic contribution of the compounds. | | |
