# Supplementary material for: Hierarchical Virtual Screening Based on Rocaglamide Derivatives to Discover New Potential Anti-Skin Cancer Agents
Source: Front Mol Biosci. 2022 Jun 2;9:836572. doi: 10.3389/fmolb.2022.836572 (PMC9201829; doi:10.3389/fmolb.2022.836572)
Supplement: Supplementary file 2 [file Table8.docx]

**Table S8:** Toxicity results obtained using the Derek software for Hypothesis 3.

| Structures | Toxicity Prediction Alert  (in human, rat and mouse) | Toxicophoric  Group | Toxicity  Alert |
| --- | --- | --- | --- |
| PC-46924673 | No Alert | — | No Alert |
| PC-17581693 | No Alert | — | No Alert |
| PC-17581549 | No Alert | — | No Alert |
| PC-46924467 | No Alert | — | No Alert |
| PC-17578428 | No Alert | — | No Alert |
| PC-17581087 | No Alert | — | No Alert |
| PC-17581154 | No Alert | — | No Alert |
| PC-46924794 | No Alert | — | No Alert |
| PC-17581927 | No Alert | — | No Alert |
| PC-17584511 | Skin Sensitization | Substituted phenol or precursor | Plausible |
| PC-17581412 | Skin Sensitization | Substituted phenol or precursor | Plausible |
| PC-46924471 | Skin Sensitization | Substituted phenol or precursor | Plausible |
| PC-17582117 | Skin Sensitization | Substituted phenol or precursor | Plausible |
| PC-17581811 | Skin Sensitization | Substituted phenol or precursor | Plausible |
| PC-17583422 | No Alert | — | No Alert |
| PC-46924739 | No Alert | — | No Alert |
| PC-44666869 | No Alert | — | No Alert |
| PC-17581798 | No Alert | — | No Alert |
| PC-44666836 | No Alert | — | No Alert |
| PC-17581900 | No Alert | — | No Alert |
| PC-3729754 | No Alert | — | No Alert |
| PC-44666755 | No Alert | — | No Alert |
| PC-17581241 | No Alert | — | No Alert |
| PC-17581023 | Skin Sensitization | Substituted phenol or precursor | Plausible |
| PC-3811421 | No Alert | — | No Alert |
| PC-3717862 | No Alert | — | No Alert |

PC: PubChem
