# Supplementary material for: Hierarchical Virtual Screening Based on Rocaglamide Derivatives to Discover New Potential Anti-Skin Cancer Agents
Source: Front Mol Biosci. 2022 Jun 2;9:836572. doi: 10.3389/fmolb.2022.836572 (PMC9201829; doi:10.3389/fmolb.2022.836572)
Supplement: Supplementary file 3 [file Table12.docx]

**Table S12:** Toxicity results obtained using the Derek software for Hypothesis 7.

| Structures | Toxicity Prediction Alert  (in human, rat and mouse) | Toxicophoric  Group | Toxicity  Alert |
| --- | --- | --- | --- |
| PC-46924665 | No Alert | — | No Alert |
| PC-17581798 | No Alert | — | No Alert |
| PC-44666869 | No Alert | — | No Alert |
| PC-3729754 | No Alert | — | No Alert |
| PC-17582683 | Skin Sensitization | Substituted phenol or precursor | Plausible |
| PC-46924722 | Skin Sensitization | Substituted phenol or precursor | Plausible |
| PC-15999896 | Skin Sensitization | Substituted phenol or precursor | Plausible |
| PC-121540950 | Skin Sensitization | Phenyl ester | Plausible |
| PC-91973461 | No Alert | — | No Alert |
| PC-46924475 | Skin Sensitization | Substituted phenol or precursor | Plausible |
| PC-46924670 | Skin Sensitization | Substituted phenol or precursor | Plausible |
| MCULE-1776053618 | No Alert | — | No Alert |
| PC-16323383 | No Alert | — | No Alert |

PC: PubChem
