# Supplementary material for: Hierarchical Virtual Screening Based on Rocaglamide Derivatives to Discover New Potential Anti-Skin Cancer Agents
Source: Front Mol Biosci. 2022 Jun 2;9:836572. doi: 10.3389/fmolb.2022.836572 (PMC9201829; doi:10.3389/fmolb.2022.836572)
Supplement: Supplementary file 4 [file Table7.docx]

**Table S7** Pharmacokinetic results obtained using the web-based application (SwissADME) for Hypothesis 7.

| Structures | MW  (<500 g/mol) | H-bond acceptors  (≤ 10) | H-bond donors  (≤5) | TPSA  (<140 A°²) | iLOGP  (≤5) | GI absorption | BBB permeant | Lipinski  Violations |
| --- | --- | --- | --- | --- | --- | --- | --- | --- |
| PC-46924665 | 488.49 | 8 | 1 | 104.68 | 2.63 | High | No | 0 |
| PC-17581798 | 488.49 | 8 | 1 | 104.68 | 2.81 | High | No | 0 |
| PC-44666869 | 488.49 | 8 | 1 | 104.68 | 2.81 | High | No | 0 |
| PC-3729754 | 498.53 | 7 | 1 | 88.89 | 3.94 | High | No | 0 |
| PC-17582683 | 458.46 | 7 | 1 | 95.45 | 2.61 | High | No | 0 |
| PC-46924722 | 458.46 | 7 | 1 | 95.45 | 2.61 | High | No | 0 |
| PC-15999896 | 491.54 | 7 | 1 | 99.22 | 3.21 | High | No | 0 |
| PC-121540950 | 497.50 | 7 | 1 | 96.22 | 3.17 | High | No | 0 |
| PC-91973461 | 483.47 | 9 | 2 | 125.69 | 3.30 | High | No | 0 |
| PC-46924475 | 458.46 | 7 | 1 | 95.45 | 03.02 | High | No | 0 |
| PC-46924670 | 458.46 | 7 | 1 | 95.45 | 2.76 | High | No | 0 |
| MCULE-1776053618 | 477.51 | 7 | 3 | 114.93 | 2.44 | High | No | 0 |
| PC-16323383 | 496.58 | 7 | 1 | 131.12 | 3.57 | High | No | 0 |

MW: Molecular weight ; TPSA: Topological Polar Surface; GI: Gastroinestinal ; BBB: Blood Brain Barrier. PC: PubChem
