# Supplementary material for: Hierarchical Virtual Screening Based on Rocaglamide Derivatives to Discover New Potential Anti-Skin Cancer Agents
Source: Front Mol Biosci. 2022 Jun 2;9:836572. doi: 10.3389/fmolb.2022.836572 (PMC9201829; doi:10.3389/fmolb.2022.836572)
Supplement: Supplementary file 5 [file Table9.docx]

**Table S9:** Toxicity results obtained using the Derek software for Hypothesis 4.

| Structures | Toxicity Prediction Alert  (in human, rat and mouse) | Toxicophoric  Group | Toxicity  Alert |
| --- | --- | --- | --- |
| PC-121540950 | Skin Sensitization | Phenyl ester | Plausible |
| PC-15994145 | No Alert | — | No Alert |
| PC-4441336 | No Alert | — | No Alert |
| PC-3739245 | No Alert | — | No Alert |
| MCULE-3536691256 | Skin Sensitization | Activated N-heterocycle | Plausible |
| PC-91822579 | No Alert | — | No Alert |
| PC-1075850 | Skin Sensitization | Substituted phenol or precursor | Plausible |
| PC-53116274 | No Alert | — | No Alert |
| PC-135909858 | No Alert | — | No Alert |
| PC-126784585 | No Alert | — | No Alert |

PC: PubChem
