# Supplementary material for: Hierarchical Virtual Screening Based on Rocaglamide Derivatives to Discover New Potential Anti-Skin Cancer Agents
Source: Front Mol Biosci. 2022 Jun 2;9:836572. doi: 10.3389/fmolb.2022.836572 (PMC9201829; doi:10.3389/fmolb.2022.836572)
Supplement: Supplementary file 6 [file Table2.docx]

| **Table S2.** Filters applied to the properties of the selected molecules (Data obtained through Molinspiration©) | | | | | | | |
| --- | --- | --- | --- | --- | --- | --- | --- |
| **Structure** | Properties | | | | | | |
|  | MW | RotBonds | LogP | TPSA | Aro | HBA | HBD |
| 1 * | 505.57 | 6 | 3.49 | 97.70 | 3 | 7 | 2 |
| 2 | 521.57 | 6 | 2.78 | 117.93 | 3 | 8 | 3 |
| 3 | 492.52 | 7 | 4.45 | 103.70 | 3 | 8 | 2 |
| 4 | 508.52 | 7 | 3.73 | 123.92 | 3 | 9 | 3 |
| 5 | 520.53 | 9 | 4.87 | 109.77 | 3 | 9 | 1 |
| 6 | 522.55 | 8 | 4.04 | 112.93 | 3 | 9 | 2 |
| 7 | 506.51 | 6 | 4.28 | 112.93 | 4 | 9 | 2 |
| 8 | 495.53 | 7 | 3.24 | 132.88 | 3 | 9 | 4 |
| 9 | 509.56 | 8 | 3.61 | 118.88 | 3 | 9 | 4 |
| 10 | 534.52 | 8 | 4.70 | 119.01 | 4 | 10 | 1 |
| 11 | 537.61 | 9 | 4.16 | 99.00 | 3 | 9 | 2 |
| 12 | 448.47 | 4 | 4.32 | 86.53 | 4 | 7 | 2 |
| 13 | 550.56 | 9 | 4.87 | 111.17 | 4 | 10 | 1 |
| 14 | 628.68 | 7 | 4.19 | 132.88 | 4 | 9 | 2 |
| 15 | 535.55 | 8 | 4.30 | 125.00 | 3 | 9 | 3 |
| Min | 448.47 | 4 | 2.78 | 86.53 | 3 | 7 | 1 |
| Max | 628.68 | 9 | 4.87 | 132.00 | 4 | 10 | 4 |

* Pivot Molecule. MW: Molecular Weight; TPSA: Topological Polar Surface Area; Aro: Aromatic ;HBA: Hidrogen Bond Acceptor; HBD: Hidrogen Bond Donnor
