# Supplementary material for: Hierarchical Virtual Screening Based on Rocaglamide Derivatives to Discover New Potential Anti-Skin Cancer Agents
Source: Front Mol Biosci. 2022 Jun 2;9:836572. doi: 10.3389/fmolb.2022.836572 (PMC9201829; doi:10.3389/fmolb.2022.836572)
Supplement: Supplementary file 7 [file DataSheet1.pdf]

Hierarchical Virtual Screening from Rocaglamide Derivatives to Discovery New Potential Anti-Skin Cancer Agents

Igor V.F. dos Santos[1,2], Rosivaldo S. Borges[1,3], Guilherme M. Silva[4,5], Lúcio R. de Lima[1,3], Ruan S. Bastos[1,3], Ryan S. Ramos[1,2], Luciane B. Silva [1,3], Carlos H. T. P. da Silva [4,5] and Cleydson B. R. dos Santos[1,2,3*]

[1] Modeling and Computational Chemistry Laboratory, Federal University of Amapá, Rod. JK, KM 02, Macapá - AP.
[2] Graduate Program in Biotechnology and Biodiversity-Network BIONORTE, Federal University of Amapá, Macapá, Brazil
[3] Graduate Program inMedicinal Chemistry and Molecular Modeling, Federal University of Pará, Belém, Brazil
[4] Computational Laboratory of Pharmaceutical Chemistry, School of Pharmaceutical Sciences of Ribeirão Preto - Universidade de São Paulo, Ribeirão Preto, Brazil
[5] Departamento de Química, Faculdade de Filosofia, Ciências e Letras de Ribeirão Preto - Universidade de São Paulo, Ribeirão Preto, Brazil

*** Correspondence:**
Cleydson Breno Rodrigues dos Santos
breno@unifap.br

SUPPLEMENTARY MATERIAL

Figure S1 Redocking validation using Chk1 (PDB ID 2CGX) and its native ligand 3D3 showing poses obtained by software (a) GOLD, (b) FRED, and (c) Dockthor. Respective RMSD values are shown in each figure. Crystallographic pose of native ligands is shown in green, while docking poses from software are in gray, purple, and light blue, respectively. Figures were prepared using Maestro.
Figure S2. 2D diagram of the interactions of the 3D3 ligand and training set compounds**.** *: Pivot Molecule
Figure S3. 2D diagram of the interactions of the promissing compounds. PC: PubChem
Figure S4. Promising compounds overlap with Rocaglamide-A (yellow). PC: PubChem.
Figure S5. Overlays of the crystallographic ligand poses (in green) with the computational poses (in red): (A) 3D3 (PDB ID 2CGX), (B) RCG (PDB ID 5ZC9) and (C) V1Y (PDB ID 6XFP).
Figure S6. Results of binding affinity of the complex (3D3), control (Roc-A) and promising compounds with Chk1 receptor (PDB ID 2CGX).
Figure S7. Results of binding affinity of the complex (RCG), control (Roc-A) and promising compounds with elF4A1-ATP receptor (PDB ID 5ZC9).
Figure S8. Results of binding affinity of the complex (V1Y), control (Roc-A) and promising compounds with BRAF receptor (PDB ID 6XFP).
Figure S9. Interactions of complexed inhibitors with amino acid residues at the respective active sites. (A) 3D3 (PDB ID 2CGX), (B) RCG (PDB ID 5ZC9) and (C) V1Y (PDB ID 6XFP).
Figure S10. Interaction of promising compounds with amino acid residues in the active site of the Chk1 receptor (PDB ID 2CGX). (A) PC-135638768, (B) PC-53093220 and (C) PC-7581023.
Figure S11. Interaction of promising compounds with amino acid residues in the active site of the elF4A1-ATP receptor (PDB ID 5ZC9). (A) PC-18582767, (B) PC-16903784 and (C) PC-53093220.
Figure S12. Interaction of promising compounds with amino acid residues in the active site of the BRAF kinase receptor (PDB ID 6XFP). (A) PC-53093220, (B) PC-7581023 and (C) PC-9115580.

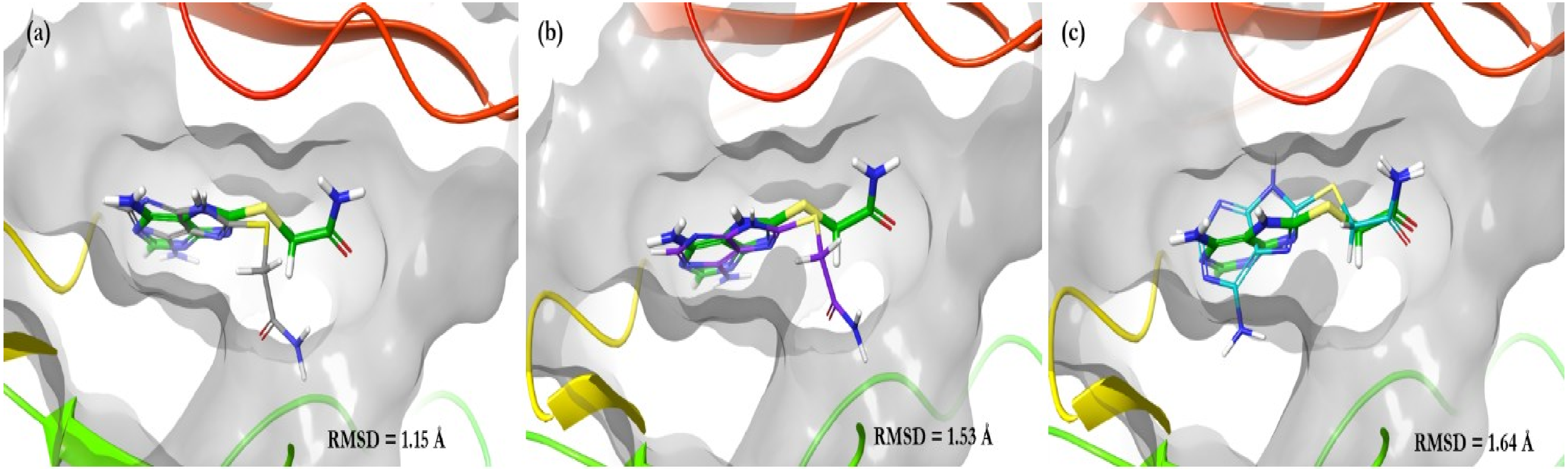

Figure S1. Redocking validation using Chk1 (PDB ID 2CGX) and its native ligand 3D3 showing poses obtained by software (a) GOLD, (b) FRED, and (c) Dockthor. Respective RMSD values are shown in each figure. Crystallographic pose of native ligands is shown in green, while docking poses from software are in gray, purple, and light blue, respectively. Figures were prepared using Maestro.

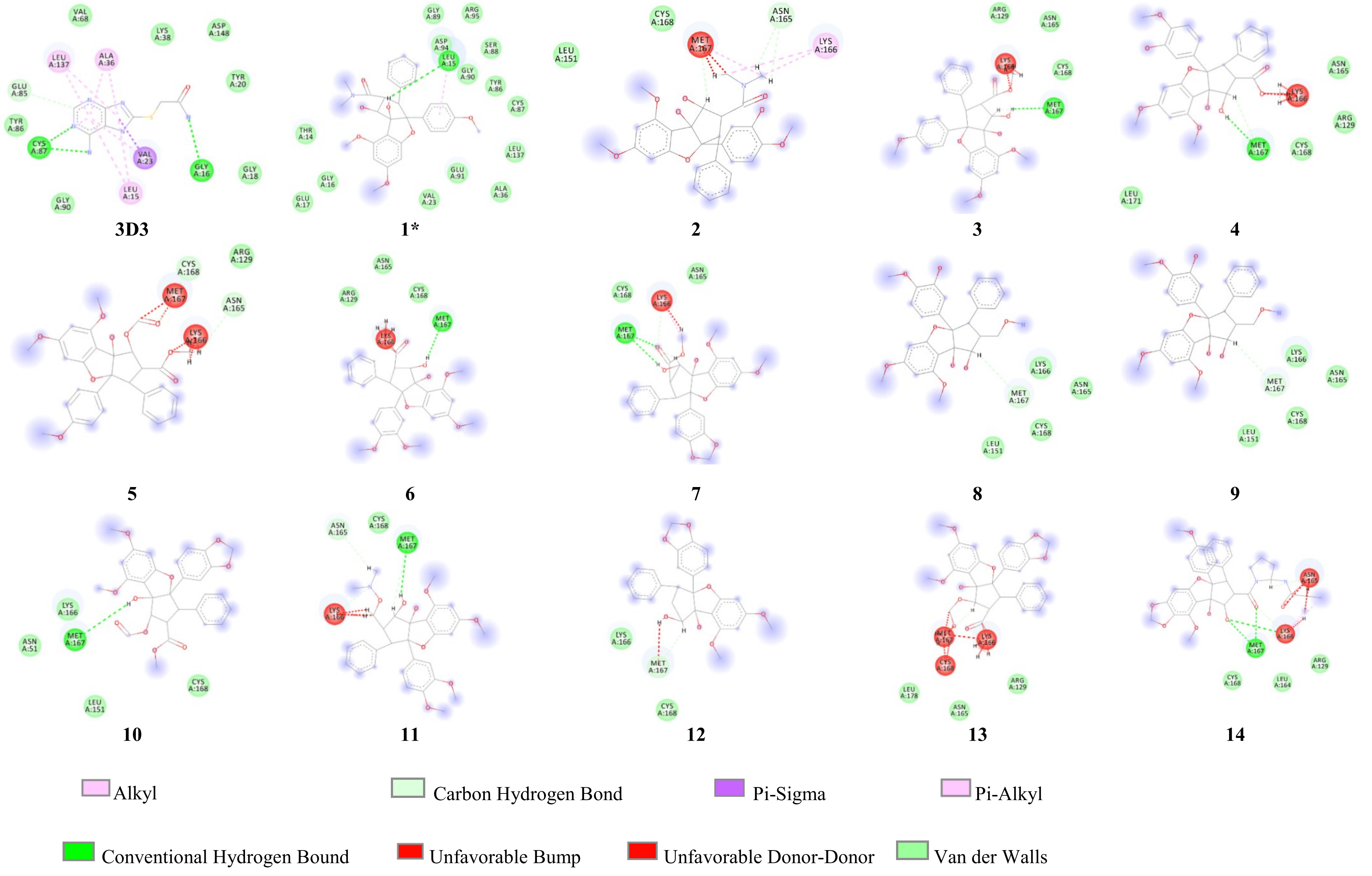

Figure S2. 2D diagram of the interactions of the 3D3 ligand and training set compounds**.** *: Pivot Molecule.

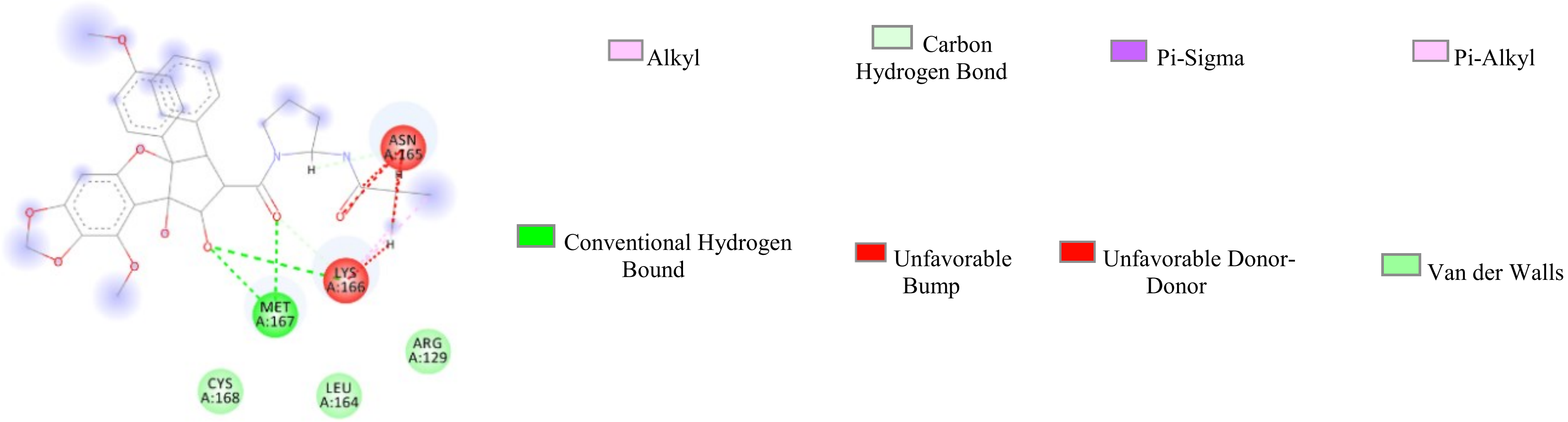

15

Figure S2 (*cont.*). 2D diagram of the interactions of the 3D3 ligand and training set compounds**.** *: Pivot Molecule

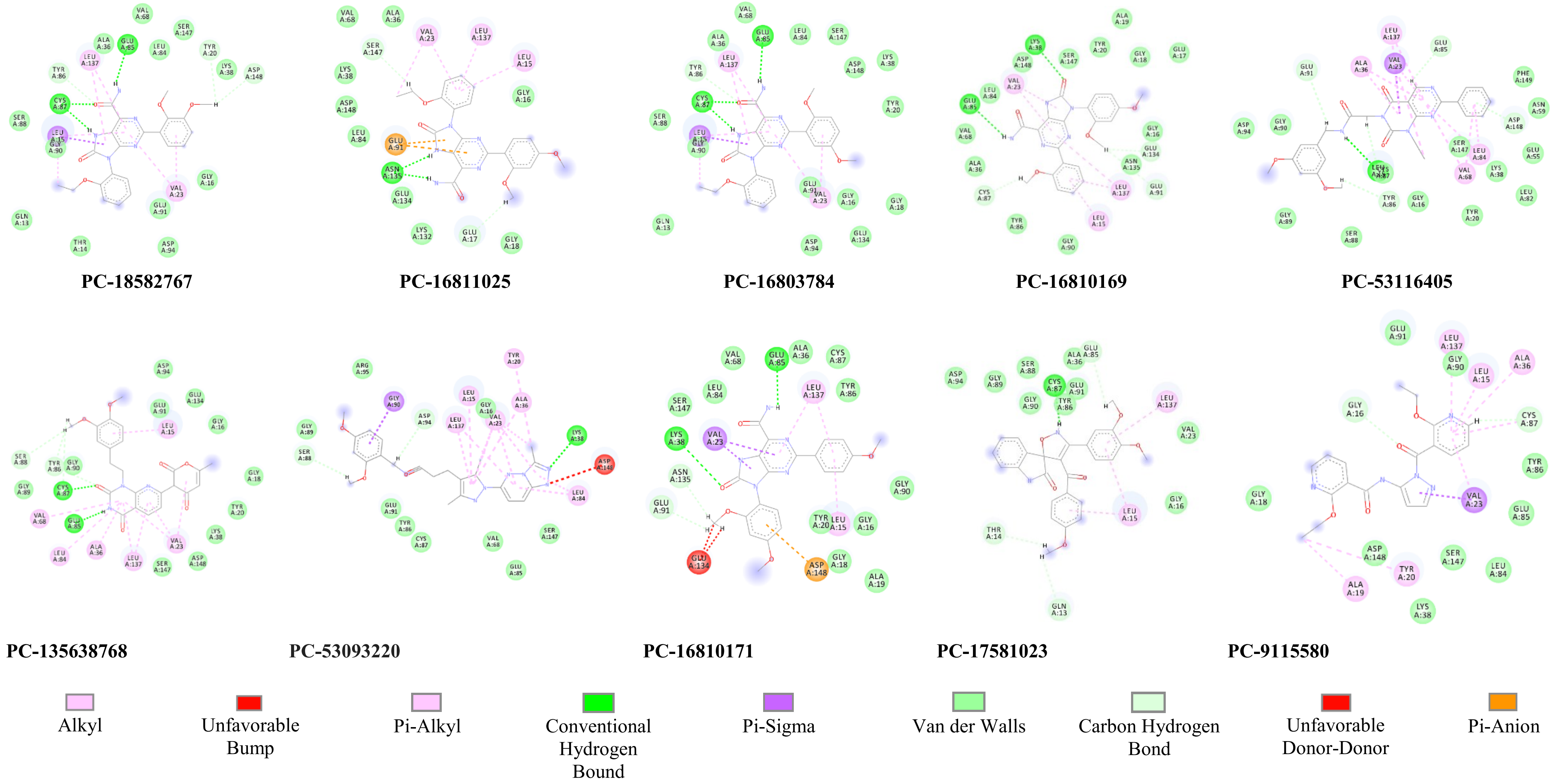

Figure S3. 2D diagram of the interactions of the promising compounds. PC: PubChem

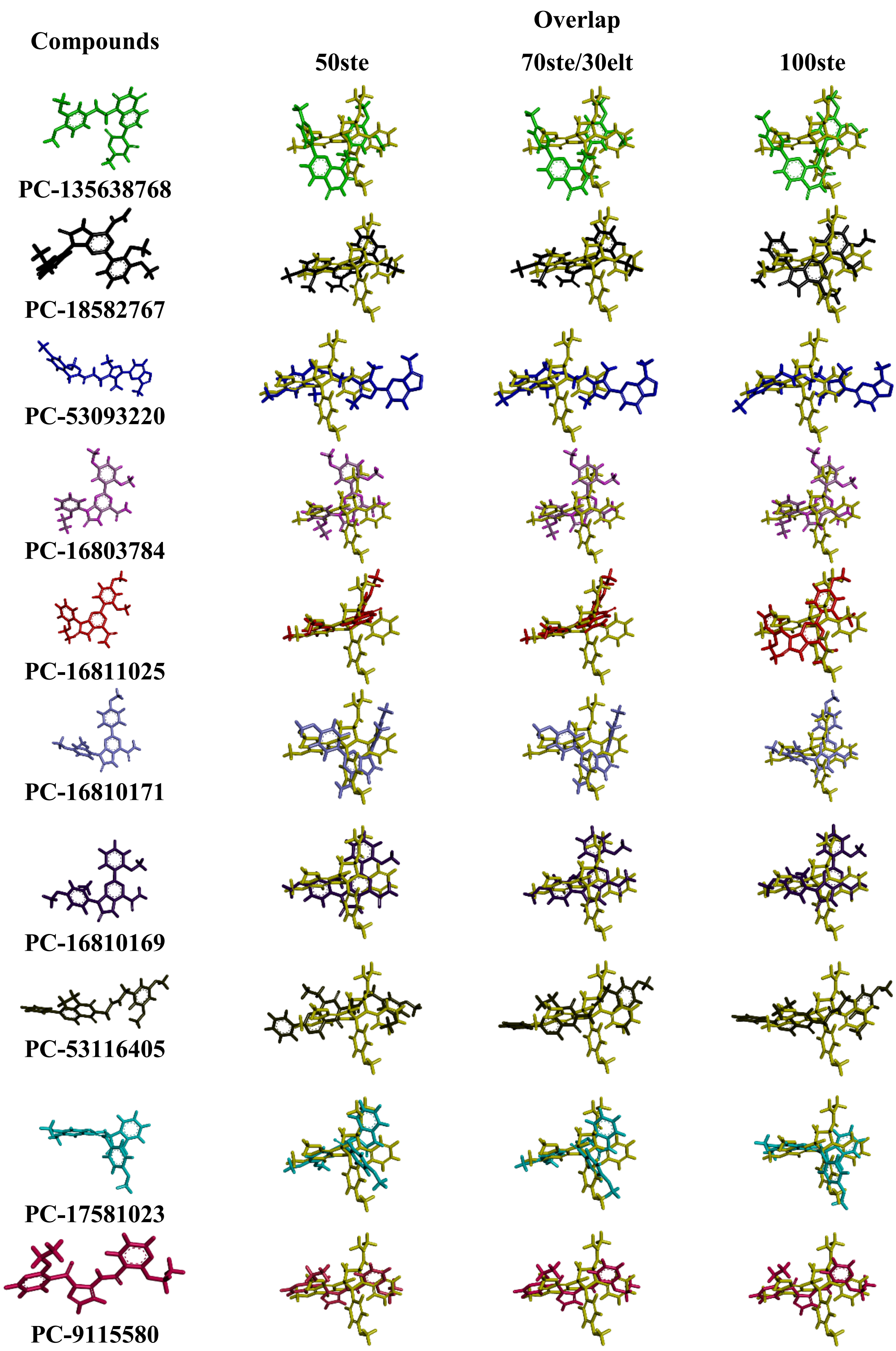

Figure S4. Promising compounds overlap with Rocaglamide-A (yellow). PC: PubChem.

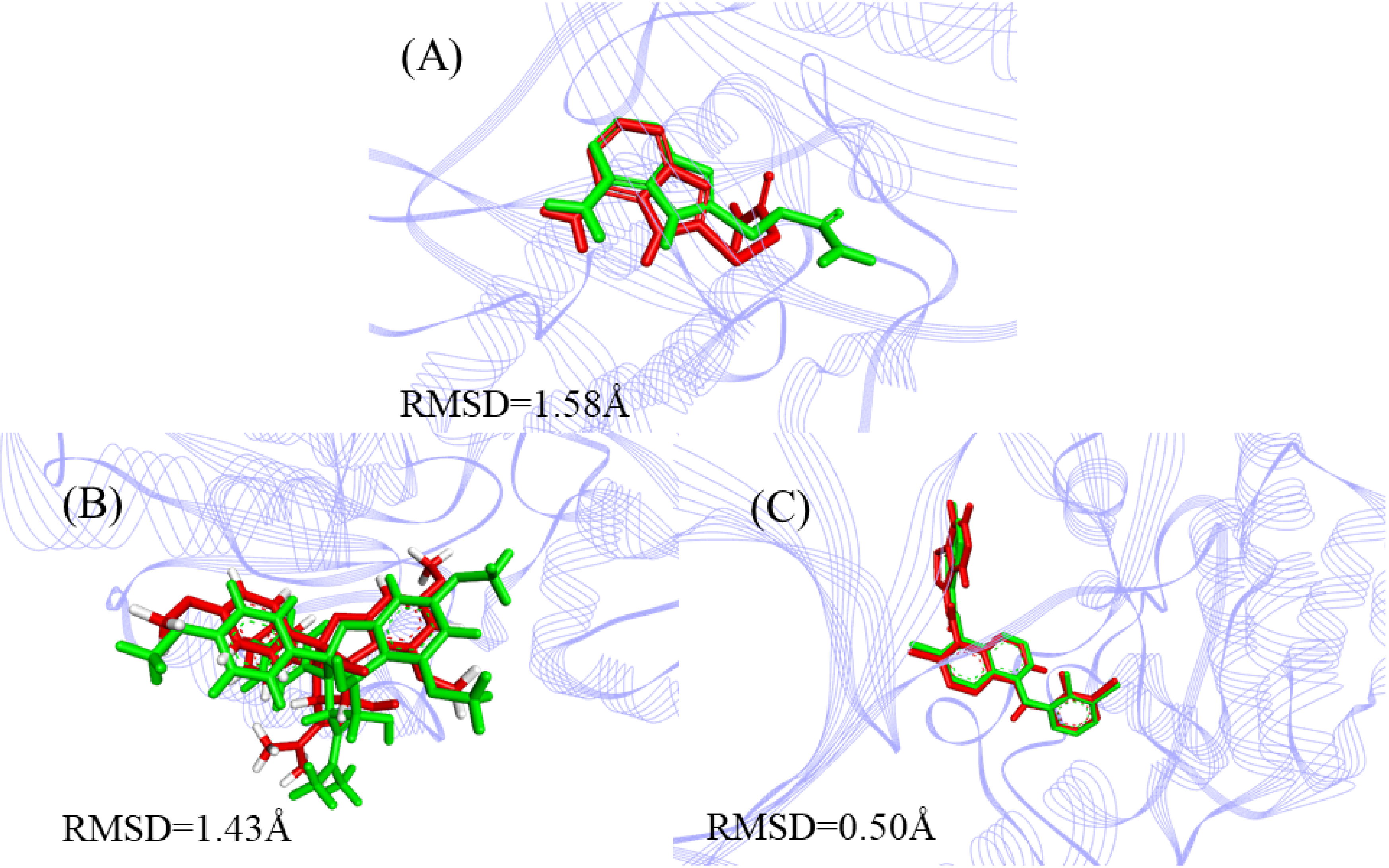

Figure S5. Overlays of the crystallographic ligand poses (in green) with the computational poses (in red): (A) 3D3 (PDB ID 2CGX), (B) RCG (PDB ID 5ZC9) and (C) V1Y (PDB ID 6XFP).

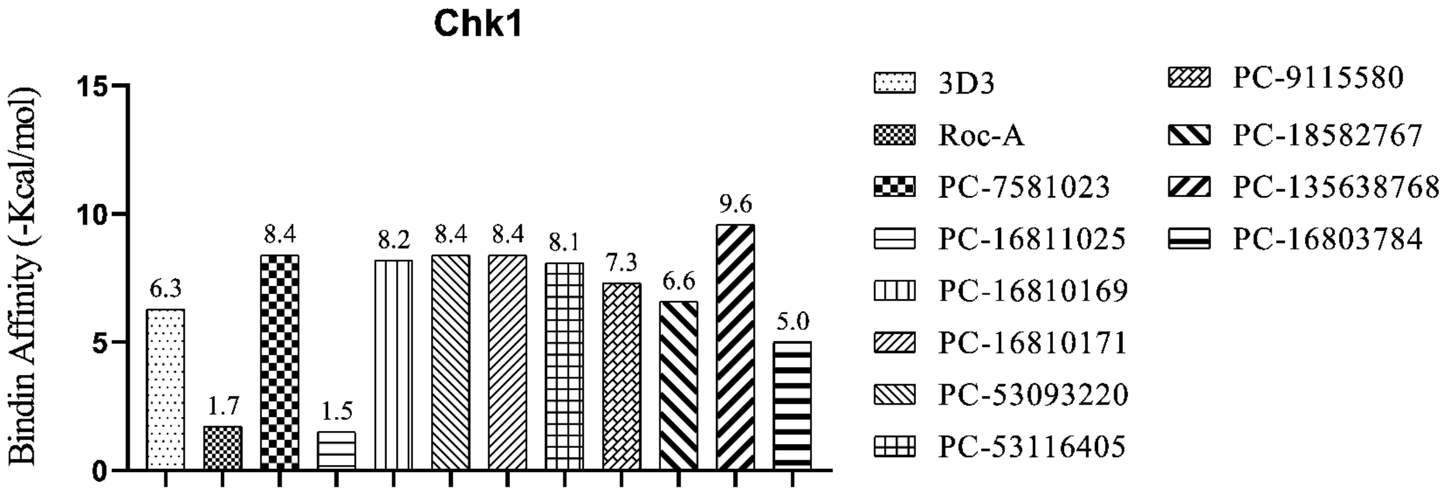

Figure S6. Results of binding affinity of the complex (3D3), control (Roc-A) and promising compounds with Chk1 receptor (PDB ID 2CGX).

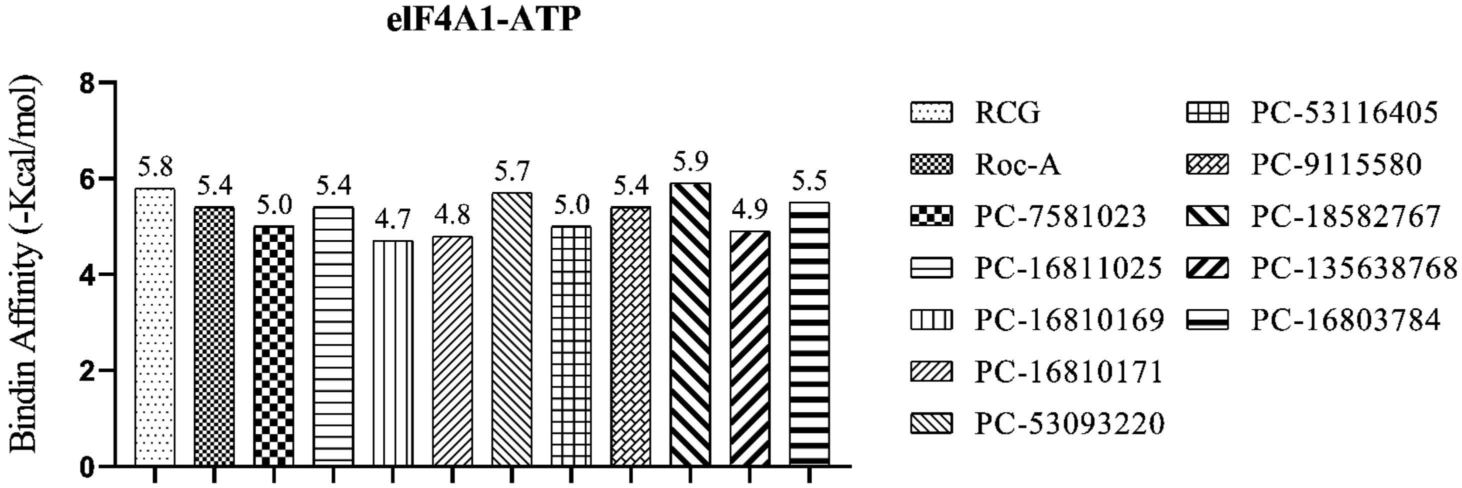

Figure S7. Results of binding affinity of the complex (RCG), control (Roc-A) and promising compounds with elF4A1-ATP receptor (PDB ID 5ZC9).

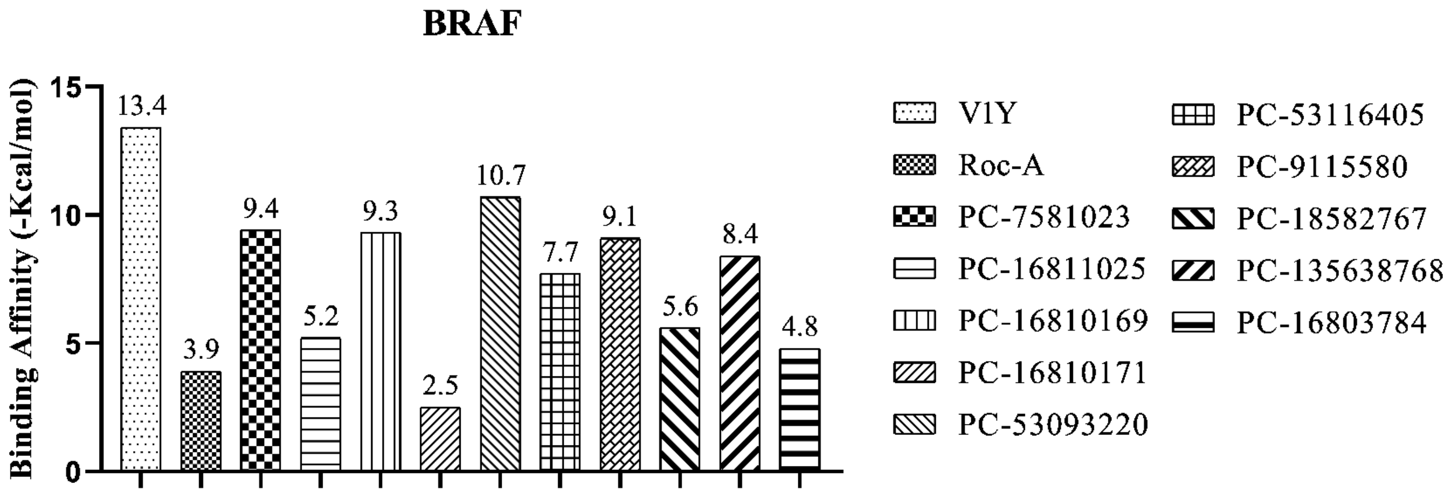

Figure S8. Results of binding affinity of the complex (V1Y), control (Roc-A) and promising compounds with BRAF receptor (PDB ID 6XFP).

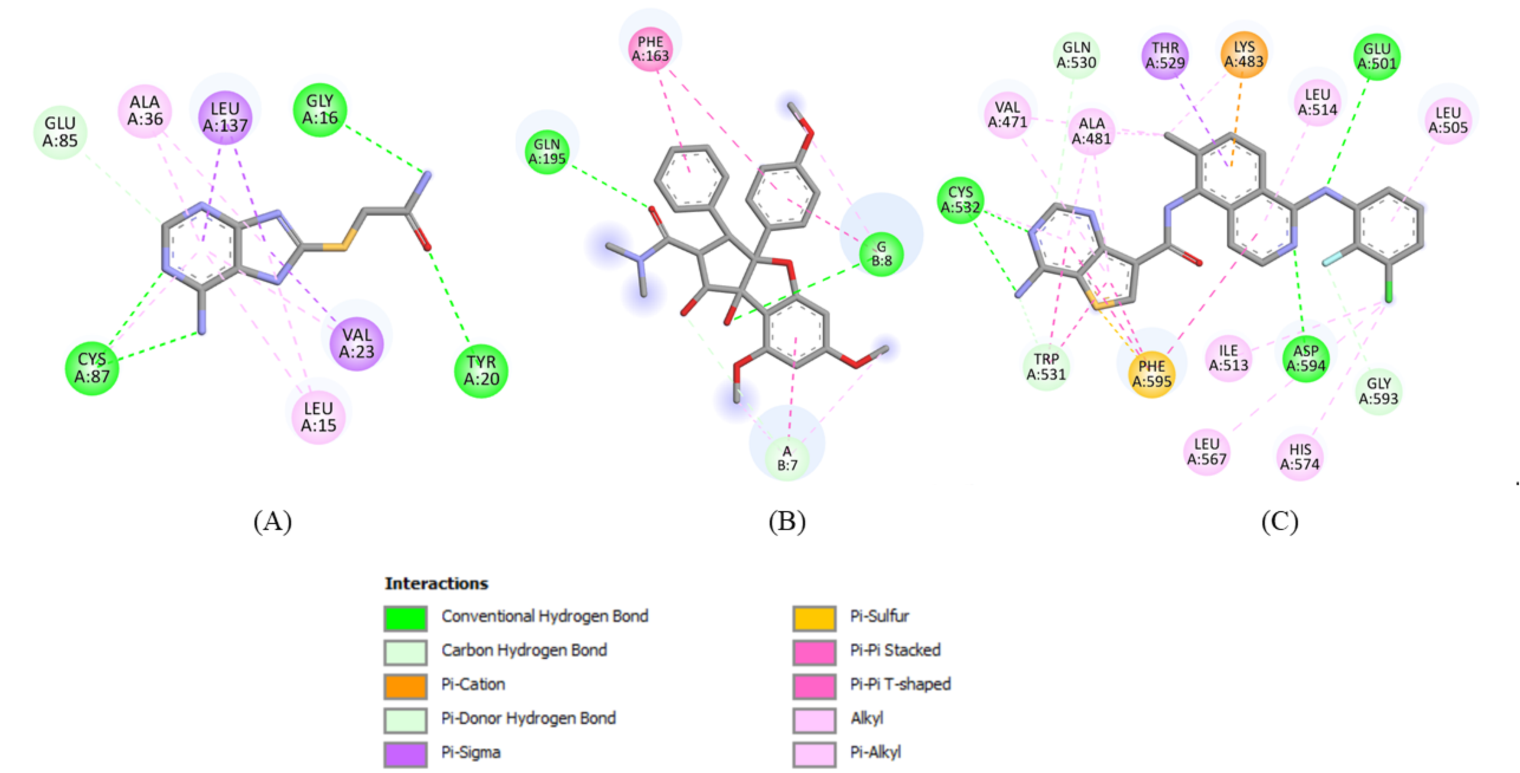

Figure S9. Interactions of complexed inhibitors with amino acid residues at the respective active sites. (A) 3D3 (PDB ID 2CGX), (B) RCG (PDB ID 5ZC9) and (C) V1Y (PDB ID 6XFP).

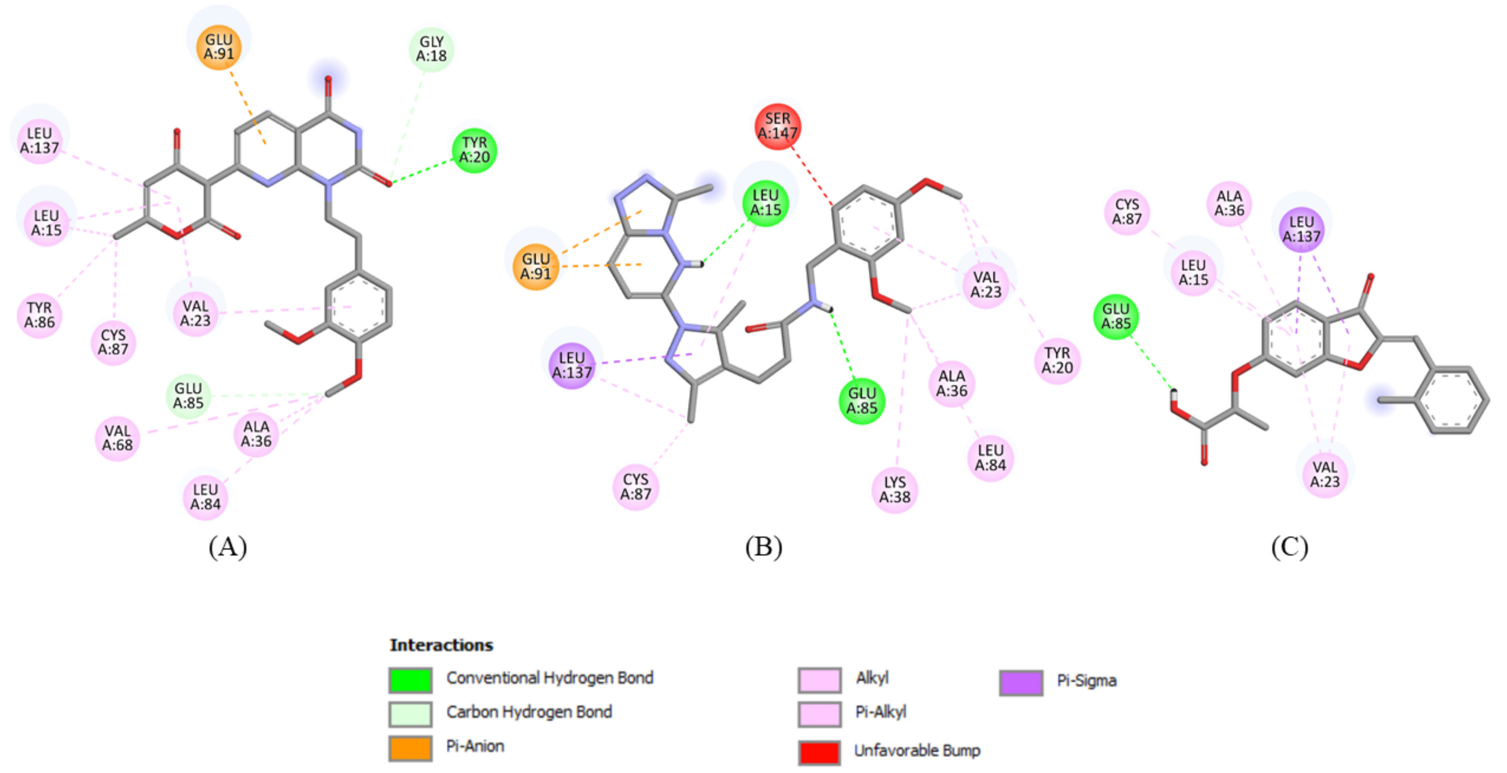

Figure S10. Interaction of promising compounds with amino acid residues in the active site of the Chk1 receptor (PDB ID 2CGX). (A) PC-135638768, (B) PC-53093220 and (C) PC-7581023.

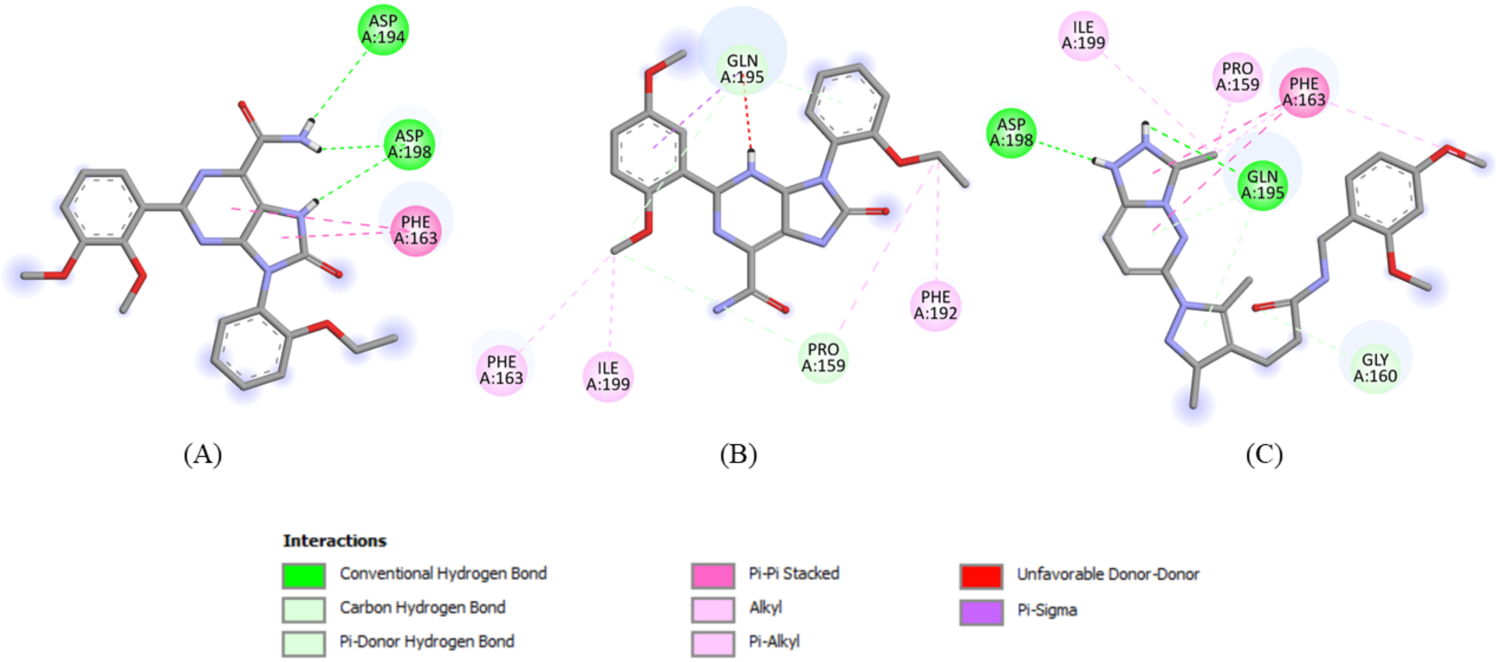

Figure S11. Interaction of promising compounds with amino acid residues in the active site of the elF4A1-ATP receptor (PDB ID 5ZC9). (A) PC-18582767, (B) PC-16903784 and (C) PC-53093220.

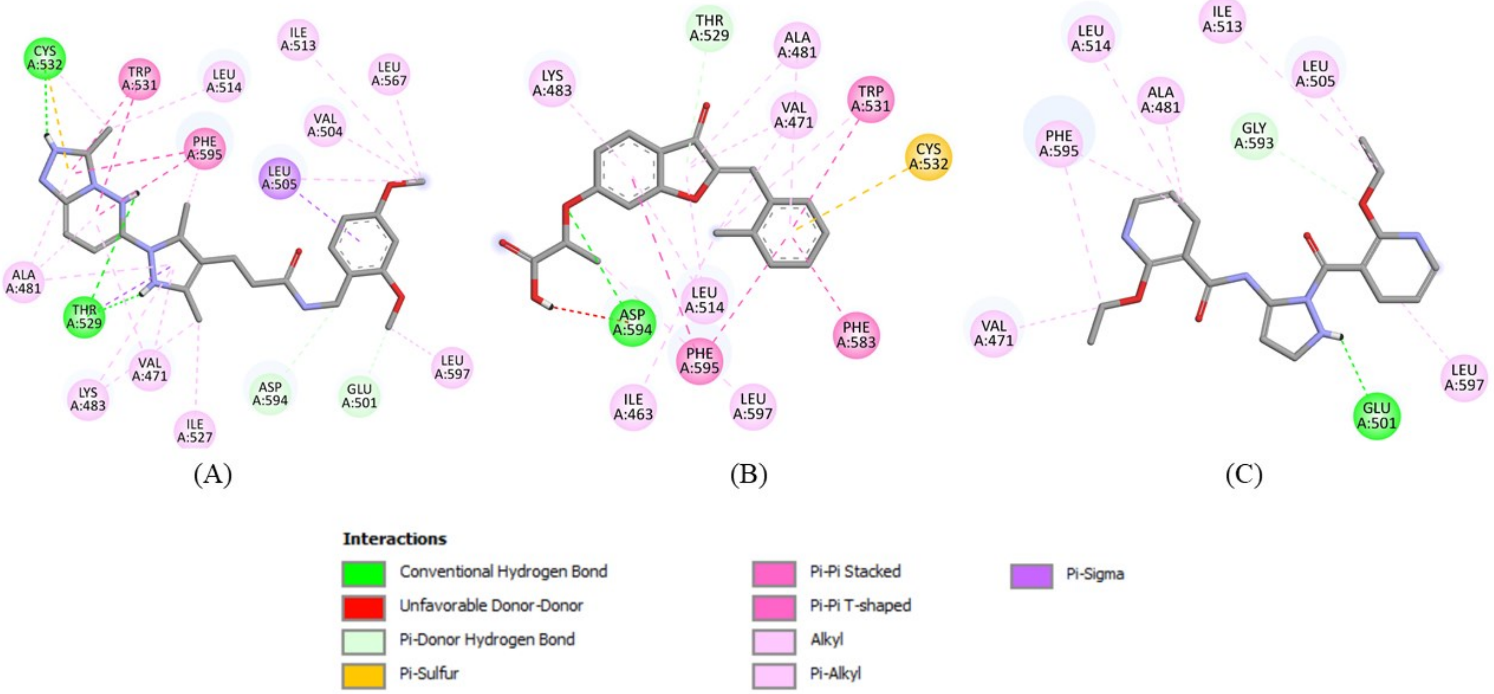

Figure S12. Interaction of promising compounds with amino acid residues in the active site of the BRAF kinase receptor (PDB ID 6XFP). (A) PC-53093220, (B) PC-7581023 and (C) PC-9115580.
