# Supplementary material for: Hierarchical Virtual Screening Based on Rocaglamide Derivatives to Discover New Potential Anti-Skin Cancer Agents
Source: Front Mol Biosci. 2022 Jun 2;9:836572. doi: 10.3389/fmolb.2022.836572 (PMC9201829; doi:10.3389/fmolb.2022.836572)
Supplement: Supplementary file 8 [file Table5.docx]

**Table S5** Pharmacokinetic results obtained using the web-based application (SwissADME) for Hypothesis 5.

| Structures | MW  (<500 g/mol) | H-bond acceptors  (≤ 10) | H-bond donors  (≤5) | TPSA  (<140 A°²) | iLOGP  (≤5) | GI absorption | BBB permeant | Lipinski  Violations |
| --- | --- | --- | --- | --- | --- | --- | --- | --- |
| PC-45172887 | 457.48 | 7 | 1 | 108.23 | 3.25 | High | No | 0 |
| PC-4871502 | 470.48 | 8 | 1 | 108.07 | 3.57 | High | No | 0 |
| PC-4896673 | 428.44 | 7 | 1 | 98.84 | 3.42 | High | No | 0 |
| MCULE-7578032479 | 466.46 | 9 | 2 | 111.23 | 3.48 | High | No | 0 |
| PC-49668561 | 445.42 | 8 | 1 | 124.97 | 2.14 | High | No | 0 |
| PC-53073532 | 441.46 | 7 | 1 | 124.97 | 2.65 | High | No | 0 |
| PC-86809391 | 427.43 | 7 | 1 | 124.97 | 2.35 | High | No | 0 |
| PC-53073422 | 461.88 | 7 | 1 | 124.97 | 2.42 | High | No | 0 |

MW: Molecular weight ; TPSA: Topological Polar Surface; GI: Gastroinestinal ; BBB: Blood Brain Barrier. PC: PubChem
