# Supplementary material for: Hierarchical Virtual Screening Based on Rocaglamide Derivatives to Discover New Potential Anti-Skin Cancer Agents
Source: Front Mol Biosci. 2022 Jun 2;9:836572. doi: 10.3389/fmolb.2022.836572 (PMC9201829; doi:10.3389/fmolb.2022.836572)
Supplement: Supplementary file 9 [file Table3.docx]

**Table S3** Pharmacokinetic results obtained using the web-based application (SwissADME) for Hypothesis 3.

| Structures | MW  (<500 g/mol) | H-bond acceptors  (≤ 10) | H-bond donors  (≤5) | TPSA  (<140 A°²) | iLOGP  (≤5) | GI absorption | BBB permeant | Lipinski  Violations |
| --- | --- | --- | --- | --- | --- | --- | --- | --- |
| PC-46924673 | 486.52 | 7 | 1 | 95.45 | 3.32 | High | No | 0 |
| PC-17581693 | 486.52 | 7 | 1 | 95.45 | 3.32 | High | No | 0 |
| PC-17581549 | 486.52 | 7 | 1 | 95.45 | 3.08 | High | No | 0 |
| PC-46924467 | 486.52 | 7 | 1 | 95.45 | 3.08 | High | No | 0 |
| PC-17578428 | 486.52 | 7 | 1 | 95.45 | 3.58 | High | No | 0 |
| PC-17581087 | 484.50 | 7 | 1 | 95.45 | 3.15 | High | No | 0 |
| PC-17581154 | 488.49 | 8 | 1 | 104.68 | 2.63 | High | No | 0 |
| PC-46924794 | 484.50 | 7 | 1 | 95.45 | 3.18 | High | No | 0 |
| PC-17581927 | 484.50 | 7 | 1 | 95.45 | 3.18 | High | No | 0 |
| PC-17584511 | 458.46 | 7 | 1 | 95.45 | 2.88 | High | No | 0 |
| PC-17581412 | 458.46 | 7 | 1 | 95.45 | 2.81 | High | No | 0 |
| PC-46924471 | 458.46 | 7 | 1 | 95.45 | 2.81 | High | No | 0 |
| PC-17582117 | 458.46 | 7 | 1 | 95.45 | 2.74 | High | No | 0 |
| PC-17581811 | 458.46 | 7 | 1 | 95.45 | 2.83 | High | No | 0 |
| PC-17583422 | 488.49 | 8 | 1 | 104.68 | 2.70 | High | No | 0 |
| PC-46924739 | 488.49 | 8 | 1 | 104.68 | 2.70 | High | No | 0 |
| PC-44666869 | 488.49 | 8 | 1 | 104.68 | 2.81 | High | No | 0 |
| PC-17581798 | 488.49 | 8 | 1 | 104.68 | 2.81 | High | No | 0 |
| PC-44666836 | 486.52 | 7 | 1 | 95.45 | 3.39 | High | No | 0 |
| PC-17581900 | 486.52 | 7 | 1 | 95.45 | 3.39 | High | No | 0 |
| PC-3729754 | 498.53 | 7 | 1 | 88.89 | 3.94 | High | No | 0 |
| PC-44666755 | 492.91 | 7 | 1 | 95.45 | 2.96 | High | No | 0 |
| PC-17581241 | 492.91 | 7 | 1 | 95.45 | 2.96 | High | No | 0 |
| PC-17581023 | 458.46 | 7 | 1 | 95.45 | 3.02 | High | No | 0 |
| PC-3811421 | 498.53 | 7 | 1 | 88.89 | 3.85 | High | No | 0 |
| PC-3717862 | 498.53 | 7 | 1 | 88.89 | 3.52 | High | No | 0 |

MW: Molecular weight ; TPSA: Topological Polar Surface; GI: Gastroinestinal ; BBB: Blood Brain Barrier. PC: PubChem
