# Supplementary material for: Hierarchical Virtual Screening Based on Rocaglamide Derivatives to Discover New Potential Anti-Skin Cancer Agents
Source: Front Mol Biosci. 2022 Jun 2;9:836572. doi: 10.3389/fmolb.2022.836572 (PMC9201829; doi:10.3389/fmolb.2022.836572)
Supplement: Supplementary file 10 [file Table13.docx]

**Table S13** Prediction of lipophilicity through the free web tool SwissADME

| Compound | iLOGP | XLOGP | WLOGP | MLOGP | SILICOS-IT Log P | Consensus Log P |
| --- | --- | --- | --- | --- | --- | --- |
| Pivotal Molecule | 3.73 | 2.83 | 2.83 | 1.55 | 3.42 | 2.87 |
| PC-18582767 | 3.36 | 2.07 | 2.29 | 1.19 | 2.76 | 2.33 |
| PC-16811025 | 3.39 | 2.07 | 2.29 | 1.19 | 2.76 | 2.34 |
| PC-16803784 | 3.36 | 2.07 | 2.29 | 1.19 | 2.76 | 2.33 |
| PC-16810171 | 3.12 | 1.70 | 1.90 | 0.97 | 2.36 | 2.01 |
| PC-135638768 | 2.90 | 2.06 | 1.98 | 1.58 | 4.00 | 2.50 |
| PC-53093220 | 3.75 | 2.19 | 2.35 | 2.60 | 2.71 | 2.72 |
| PC-16810169 | 3.27 | 1.70 | 1.90 | 0.97 | 2.36 | 2.04 |
| PC-17581023 | 3.19 | 3.81 | 2.73 | 1.63 | 4.57 | 3.19 |
| PC-9115580 | 3.35 | 2.70 | 2.22 | 1.75 | 1.78 | 2.36 |
| PC-53116405 | 3.41 | 2.23 | 1.82 | 1.32 | 2.98 | 2.35 |

PC: PubChem
