# Supplementary material for: Hierarchical Virtual Screening Based on Rocaglamide Derivatives to Discover New Potential Anti-Skin Cancer Agents
Source: Front Mol Biosci. 2022 Jun 2;9:836572. doi: 10.3389/fmolb.2022.836572 (PMC9201829; doi:10.3389/fmolb.2022.836572)
Supplement: Supplementary file 11 [file Table4.docx]

**Table S4** Pharmacokinetic results obtained using the web-based application (SwissADME) for Hypothesis 4.

| Structures | MW  (<500 g/mol) | H-bond acceptors  (≤ 10) | H-bond donors  (≤5) | TPSA  (<140 A°²) | iLOGP  (≤5) | GI absorption | BBB permeant | Lipinski  Violations |
| --- | --- | --- | --- | --- | --- | --- | --- | --- |
| PC-121540950 | 497.50 | 7 | 1 | 96.22 | 3.17 | High | No | 0 |
| PC-15994145 | 477.51 | 7 | 1 | 99.22 | 3.31 | High | No | 0 |
| PC-4441336 | 453.44 | 7 | 2 | 119.28 | 3.21 | High | No | 0 |
| PC-3739245 | 477.51 | 7 | 1 | 114.90 | 3.64 | High | No | 0 |
| MCULE-3536691256 | 408.45 | 7 | 1 | 106.26 | 3.39 | High | No | 0 |
| PC-91822579 | 454.54 | 7 | 1 | 109.59 | 3.79 | High | No | 0 |
| PC-1075850 | 484.52 | 7 | 1 | 111.78 | 3.27 | High | No | 0 |
| PC-53116274 | 495.91 | 7 | 1 | 117.34 | 3.89 | High | No | 0 |
| PC-135909858 | 425.46 | 7 | 1 | 129.45 | 2.70 | High | No | 0 |
| PC-126784585 | 390.40 | 7 | 1 | 120.48 | 2.45 | High | No | 0 |

MW: Molecular weight ; TPSA: Topological Polar Surface; GI: Gastroinestinal ; BBB: Blood Brain Barrier. PC: PubChem
