# Supplementary material for: Hierarchical Virtual Screening Based on Rocaglamide Derivatives to Discover New Potential Anti-Skin Cancer Agents
Source: Front Mol Biosci. 2022 Jun 2;9:836572. doi: 10.3389/fmolb.2022.836572 (PMC9201829; doi:10.3389/fmolb.2022.836572)
Supplement: Supplementary file 12 [file Table11.docx]

**Table S11:** Toxicity results obtained using the Derek software for Hypothesis 6.

| Structures | Toxicity Prediction Alert  (in human, rat and mouse) | Toxicophoric  Group | Toxicity  Alert |
| --- | --- | --- | --- |
| PC-135646199 | No Alert | — | No Alert |
| MCULE-8528252019 | No Alert | — | No Alert |
| PC-13564619 | No Alert | — | No Alert |
| MCULE-2173262466 | Skin Sensitization | Substituted phenol or precursor | Plausible |
| PC-127253824 | No Alert | — | No Alert |
| PC-91820937 | No Alert | — | No Alert |
| PC-20869621 | No Alert | — | No Alert |
| PC-70748120 | No Alert | — | No Alert |
| PC-16806650 | No Alert | — | No Alert |
| MCULE-6895478295 | No Alert | — | No Alert |
| MCULE-5562691993 | No Alert | — | No Alert |
| PC-9115580 | No Alert | — | No Alert |

PC: PubChem
