# Supplementary material for: Hierarchical Virtual Screening Based on Rocaglamide Derivatives to Discover New Potential Anti-Skin Cancer Agents
Source: Front Mol Biosci. 2022 Jun 2;9:836572. doi: 10.3389/fmolb.2022.836572 (PMC9201829; doi:10.3389/fmolb.2022.836572)
Supplement: Supplementary file 13 [file Table6.docx]

**Table S6** Pharmacokinetic results obtained using the web-based application (SwissADME) for Hypothesis 6.

| Structures | MW  (<500 g/mol) | H-bond acceptors  (≤ 10) | H-bond donors  (≤5) | TPSA  (<140 A°²) | iLOGP  (≤5) | GI absorption | BBB permeant | Lipinski  Violations |
| --- | --- | --- | --- | --- | --- | --- | --- | --- |
| PC-135646199 | 433.46 | 7 | 1 | 100.39 | 2.85 | High | No | 0 |
| MCULE-5007521800 | 455.48 | 7 | 2 | 103.26 | 0.57 | High | No | 0 |
| PC-135646197 | 433.46 | 7 | 1 | 100.39 | 2.82 | High | No | 0 |
| MCULE-2173262466 | 429.38 | 9 | 2 | 110.86 | 2.27 | High | No | 0 |
| PC-127253824 | 488.49 | 8 | 1 | 125.55 | 3.41 | High | No | 0 |
| PC-91820937 | 440.52 | 7 | 1 | 109.59 | 3.45 | High | No | 0 |
| PC-20869621 | 452.44 | 8 | 1 | 113.16 | 3.19 | High | No | 0 |
| PC-70748120 | 368.35 | 8 | 3 | 127.19 | 1.98 | High | No | 0 |
| PC-16806650 | 468.89 | 7 | 1 | 113.16 | 3.31 | High | No | 0 |
| MCULE-6895478295 | 370.38 | 7 | 1 | 124.66 | 2.24 | High | No | 0 |
| MCULE-5562691993 | 416.39 | 8 | 2 | 138.10 | 1.62 | High | No | 0 |

MW: Molecular weight ; TPSA: Topological Polar Surface; GI: Gastroinestinal ; BBB: Blood Brain Barrier. PC: PubChem
