# Supplementary material for: Hierarchical Virtual Screening Based on Rocaglamide Derivatives to Discover New Potential Anti-Skin Cancer Agents
Source: Front Mol Biosci. 2022 Jun 2;9:836572. doi: 10.3389/fmolb.2022.836572 (PMC9201829; doi:10.3389/fmolb.2022.836572)
Supplement: Supplementary file 14 [file Table14.docx]

**Table S14** Prediction of solubility through the free web tool SwissADME

| Compound | ESOL | ALI | SILICOS-IT Log S | Consensus Log S |
| --- | --- | --- | --- | --- |
| Pivotal Molecule | -4.66 | -4.54 | -6.95 | -5.38 |
| PC-18582767 | -3.87 | -4.52 | -6.55 | -4.98 |
| PC-16811025 | -3.87 | -4.52 | -6.55 | -4.98 |
| PC-16803784 | -3.87 | -4.52 | -6.55 | -4.98 |
| PC-16810171 | -3.63 | -4.14 | -6.16 | -4.64 |
| PC-135638768 | -4.03 | -4.56 | -7.27 | -5.29 |
| PC-53093220 | -3.86 | -4.10 | -6.90 | -4.95 |
| PC-16810169 | -3.63 | -4.14 | -6.16 | -4.64 |
| PC-17581023 | -5.08 | -5.51 | -8.20 | -6.26 |
| PC-9115580 | -3.76 | -4.63 | -5.65 | -4.68 |
| PC-53116405 | -4.06 | -4.33 | -7.49 | -5.29 |

PC: PubChem
