# Supplementary material for: Hierarchical Virtual Screening Based on Rocaglamide Derivatives to Discover New Potential Anti-Skin Cancer Agents
Source: Front Mol Biosci. 2022 Jun 2;9:836572. doi: 10.3389/fmolb.2022.836572 (PMC9201829; doi:10.3389/fmolb.2022.836572)
Supplement: Supplementary file 15 [file Table10.docx]

**Table S10:** Toxicity results obtained using the Derek software for Hypothesis 5.

| Structures | Toxicity Prediction Alert  (in human, rat and mouse) | Toxicophoric  Group | Toxicity  Alert |
| --- | --- | --- | --- |
| PC-45172887 | Skin Sensitization | Hydrazine or precursor | Plausible |
| PC-4871502 | Skin Sensitization | Substituted phenol or precursor | Plausible |
| PC-4896673 | Skin Sensitization | Substituted phenol or precursor | Plausible |
| MCULE-7578032479 | Skin Sensitization | Imine or alpha,beta-unsaturated imine | Plausible |
| PC-49668561 | Skin Sensitization | Hydrazine or precursor | Plausible |
| PC-53073532 | Skin Sensitization | Hydrazine or precursor | Plausible |
| PC-86809391 | Skin Sensitization | Hydrazine or precursor | Plausible |
| PC-53073422 | Skin Sensitization | Hydrazine or precursor | Plausible |

PC: PubChem
